# Flexible ultrasound transceiver array for non-invasive surface-conformable imaging enabled by geometric phase correction

**DOI:** 10.1038/s41598-022-20721-7

**Published:** 2022-09-28

**Authors:** Jeffrey Elloian, Jakub Jadwiszczak, Volkan Arslan, Jeffrey D. Sherman, David O. Kessler, Kenneth L. Shepard

**Affiliations:** 1 Department of Electrical Engineering, Columbia University, 500 W 120th St., New York, NY 10027 USA; 2 Department of Biomedical Engineering, Columbia University, 1210 Amsterdam Avenue, New York, NY 10027 USA; 3 Department of Emergency Medicine, Morgan Stanley Children’s Hospital of New York Presbyterian at Columbia University Medical Center, New York, 10032 USA

**Keywords:** Biomedical engineering, Electrical and electronic engineering

## Abstract

Ultrasound imaging provides the means for non-invasive real-time diagnostics of the internal structure of soft tissue in living organisms. However, the majority of commercially available ultrasonic transducers have rigid interfaces which cannot conform to highly-curved surfaces. These geometric limitations can introduce a signal-quenching air gap for certain topographies, rendering accurate imaging difficult or impractical. Here, we demonstrate a 256-element flexible two-dimensional (2D) ultrasound piezoelectric transducer array with geometric phase correction. We show surface-conformable real-time B-mode imaging, down to an extreme radius of curvature of 1.5 cm, while maintaining desirable performance metrics such as high signal-to-noise ratio (SNR) and minimal elemental cross-talk at all stages of bending. We benchmark the array capabilities by resolving reflectors buried at known locations in a medical-grade tissue phantom, and demonstrate how phase correction can improve image reconstruction on curved surfaces. With the current array design, we achieve an axial resolution of ≈ 2 mm at clinically-relevant depths in tissue, while operating the array at 1.4 MHz with a bandwidth of ≈ 41%. We use our prototype to image the surface of the human humerus at different positions along the arm, demonstrating proof-of-concept applicability for real-time diagnostics using phase-corrected flexible ultrasound probes.

## Introduction

Diagnostic ultrasound has become an invaluable imaging modality to healthcare professionals for the purpose of non-invasive examination of soft tissue, as it carries with it no associated risks of exposure to cell-damaging ionising radiation. This method of non-destructive imaging is usually achieved with linear arrays of piezoelectric ultrasound transducers, which convert mechanical sound energy to electrical signals and vice versa^1^. The majority of commercially available transducers are housed in a rigid probe and have fixed interfaces that cannot conform to the surface of the human body in three dimensions. Thus, many clinical incentives exist to create flexible, body-conformable ultrasound probes that can match the frequency and resolution performance of contemporary rigid-interface arrays for biomedical applications.

2D phased arrays possess the additional advantage of steering the generated ultrasound beam in three dimensions at arbitrary focal lengths, which can be used for detailed localized imaging^2,3^. Among many imaging applications, ultrasound has been demonstrated as a viable modality to help diagnose bone fractures and dynamically guide fracture reduction—especially useful in low resource settings as an alternative to X-rays. The non-ionising nature of ultrasound is particularly important when examining fractures in minors, where there is a strong desire to avoid unnecessary exposure to X-rays^4^. In a study of 224 suspected fractures in children, 86.6% of the cases were correctly identified using ultrasound^5^. Ultrasound also provided the highest sensitivity for identifying fractures in long bones, specifically the humerus, with a sensitivity as high as 98% across 233 individuals^6^. The diameter of an adult humerus in the midshaft position varies statistically from 17 to 23 mm for males, and 14–20 mm for females^7^, comfortably resolvable by ultrasonic frequencies below 1 MHz. Development of flexible ultrasound phased arrays, as described in this paper, may thus provide a convenient solution for safe, rapid, and accurate detection of fractures in the upper limbs, as well as other musculoskeletal abnormalities; removing the dependence on the expertise of the operator. Flexible probes for these and other similar applications must, at the smallest extent, bend to conform to a cylinder with a radius of curvature of ~ 1.5 cm to ensure accurate image reconstruction.

In general, the concept of creating flexible or stretchable ultrasound arrays involves the direct integration of ultrasound transducer elements onto a flexible interconnect substrate. Prior efforts in this area have generally integrated bulk samples of the 5H phase of lead zirconate titanate (PZT-5H) or piezo-composites onto a polymer backing substrate with electrodes for the signal and ground connections^8–10^. A notable advance in the field was the introduction of a stretchable patch containing 100 separate 1–3 piezo-composite elements, arranged in a 2D array on a stretchable polydimethylsiloxane (PDMS) substrate^11–13^. One-dimensional (1D) wrap-around arrays have also been demonstrated, but they typically operate with 8–128 transducer elements^14^. To maintain good ultrasound array flexibility, the presence of backing/matching layers and/or kerf fill material between array elements often needs to be sacrificed. This results in decreased control of the ultrasound waveform, but the associated trade-offs on the usual figures of merit have not been defined comprehensively in the literature for these novel flexible ultrasound arrays. Many of these systems have technical shortcomings, such as too wide a pitch between elements, leading to the formation of grating lobes at the designated frequencies of operation, and no consideration for array curvature and its effect on phase interference; ultimately degrading the performance of the imaging system by introducing errors and artefacts in the final image.

Here, we demonstrate the FlexArray—a mechanically flexible, passive, and phased 2D ultrasound array containing 256 piezoelectric transducers integrated directly onto a flexible printed circuit board (PCB), which utilises geometric phase correction to compensate for the changing radius of curvature when imaging curved objects. We describe the fabrication process and functionality of the array and discuss the phasing algorithm employed for image correction. Using COMSOL Multiphysics simulations, we address device design considerations for achieving good array flexibility and the potential cost of fabrication ease and/or ultrasound performance by comparing different kerf fill materials. We demonstrate the imaging performance of the conformable array on phantom soft matter of various shapes, with and without phase correction. The FlexArray demonstrates controllable beam steering functionality and operational pressures of > 500 kPa at the focal point, at a resonant driving frequency of ~ 1.4 MHz. The piezoelectric transducer elements have a wide bandwidth (41.3%), making them good candidates for interfacing and interrogating biological tissue. The cross-talk between neighbouring elements is suitably low (~ − 52 dB to the first neighbour, ~ − 74 dB to the second neighbour), allowing for high-contrast imaging with minimal electrical interference. Finally, we use a prototype device to image the cross-section of the human upper arm, demonstrating the capability of this design paradigm for the development of non-invasive, non-ionising biomedical imaging apparatus that can examine bone topology.

## Results

### Device fabrication and benchmarking

**Figure 1:**
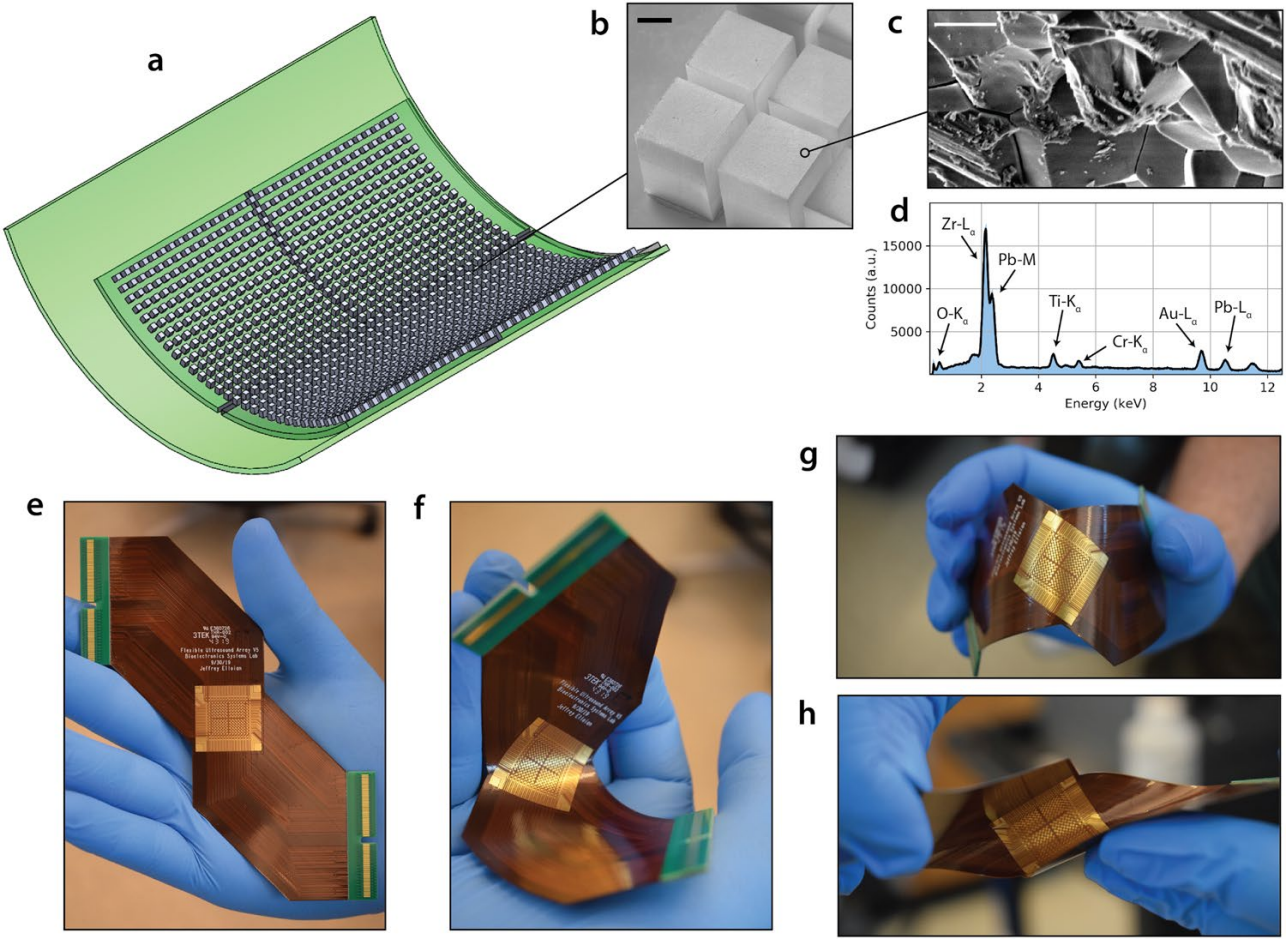
Overview of the FlexArray. (**a**) 3D rendering of the flexible array with mounted piezoelectric transducers. (**b**) Scanning electron micrograph of diced PZT pillars on the edge of an array quadrant. The pillars are contacted with a Cr/Au top metal layer. Scale bar, 200 μm. (**c**) Scanning electron micrograph presenting the surface morphology of the gold-covered PZT crystals. Scale bar, 2 μm. (**d**) Energy dispersive X-ray spectrum of the pillar, demonstrating the elemental content of the fabricated transducer. (**e**) The as-manufactured flexible PCB used to house the piezoelectric array in the center, seen held in a neutral position. (**f**) The diagonal connector design allows for the easy bending of the array by hand. The board is flexible enough to also support (**g**) convex bending, and (**h**) shear bending.

Our flexible ultrasound array utilises bulk piezoelectric transducers; each fabricated from a single pre-poled piezoelectric PZT crystal coupling directly to the vibrating medium to be imaged. When operated in thickness mode, these transducers have their height optimised to λ/2^15,16^, where λ is the wavelength at the ultrasound carrier frequency. We choose to use bulk transducers, rather than capacitive micromachined ultrasonic transducers (CMUTs) or piezoelectric micromachined ultrasonic transducers (PMUTs) for ease of fabrication and, in the case of CMUTs, to avoid requirements for a DC bias voltage^16^. In addition, for CMUTs, the longitudinal modes can also cause significant cross-talk between elements, reducing the resolution. Figure 1a shows the bulk piezoelectric PZT transducer array mounted on a flexible PCB. The FlexArray is fabricated by lithographic patterning and dicing of a single block of PZT into four square quadrants each containing 8 × 8 pillars, with individual pillars measuring 825 μm × 825 μm in area and 1.006 mm in height. The use of a thickness of ~ 0.31λ is acceptable here because the substrate alters the location of the resonance. The choice of the center frequency (~ 1.4 MHz) was determined, in part, by routing constraints for the 2D array, which limit the pixel pitch to 1000 μm, which corresponds to 0.91λ. At this pitch, grating lobes restrict the viewing area to ± 39°. Operating at a higher frequency at this pitch would therefore further narrow the field of view, limiting the imaging capability of the device.

The detailed descriptions of the fabrication process for the transducer array are included in Supplementary section 1. The scanning electron micrograph in Fig. 1b shows a corner of the metallised array after dicing, while Fig. 1c presents the typical surface morphology of the PZT pillar after coating with the top electrode metal layer of Cr/Au. Energy-dispersive X-ray spectroscopy was used to confirm the elemental content of PZT in the fabricated pillars (Fig. 1d), while readings of the piezoelectric coefficient (*d*_*33*_) indicate average values of 534 pC m^−1^ (see Supplementary Fig. S3) that are not affected by the steps in fabrication processing. We note here that as the bulk transducers are operating in longitudinal mode, the array is fabricated such that the PZT-5H poling direction is out of the plane of the device^17^.

The design, manufacturing and composition of the flexible PCB are described in Supplementary section 2. The final thickness of the array is approximately 0.26 mm in bending regions and 1.26 mm at the piezoelectric pillar locations. Figure 1e–h demonstrate several bending modalities for the as-fabricated substrate. A diagonal routing design allows the device to be conformably wrapped around the imaging target, with the exposed electrical contacts for the piezoelectric array located in the centre of the board. The minimum radius of curvature (*R*) supported by the array before the adjacent pillars on the mounted array touch is *R* > 1.4 cm, meeting the geometrical requirements to image most human body parts. The bonding process that combines the PCB substrate and the diced PZT array is carried out using standard semiconductor industry fabrication techniques (see Supplementary section 1).

We comment now on some more specific design considerations. At the low operating frequencies used here, the thin flexible PCB is effectively acoustically transparent and the material on the back side of the array acts as a backing layer, i.e., water in the case of hydrophone experiments, and air in the case of imaging experiments. We choose not to fabricate a matching layer to preserve maximum flexibility for the array—similar to other recent work on flexible ultrasound transducer arrays^11–13^. Sacrificing the matching layer decreases SNR, but it does not affect the final lateral and axial resolutions. Another consideration is the inclusion of kerf fill materials that are often used to match the acoustic impedance of piezo-ceramic elements to that of water/tissue. Epoxies are often used in 1–3 composites with PZT to decrease the acoustic impedance of the transducer. However, this kerf fill mechanically stiffens the array and limits flexibility. Not having kerf fill material—or using water or air as the kerf fill—increases the electromechanical coupling coefficient of PZT^18^. This is reflected in our own experimental data in Fig. S3, where the measured *d*_*33*_ values are shown to reliably increase after the dicing step is carried out on the PZT sheets—effectively inserting air kerf fill into the array which improves the electromechanical coupling. To verify this, we carried out Multiphysics COMSOL simulations of our transducer stack in Supplementary Fig. 4. A direct comparison between using polyimide (a soft, epoxy-like material), air and water for the kerf fill shows that the bandwidth of the response around resonance is reduced, while the acoustic pressure generated in water remains of the same order of magnitude. Thus, the use of a kerf-less array is justified here to achieve maximum array flexibility, without significantly sacrificing the electromechanical coupling of ultrasound into water.

**Figure 2:**
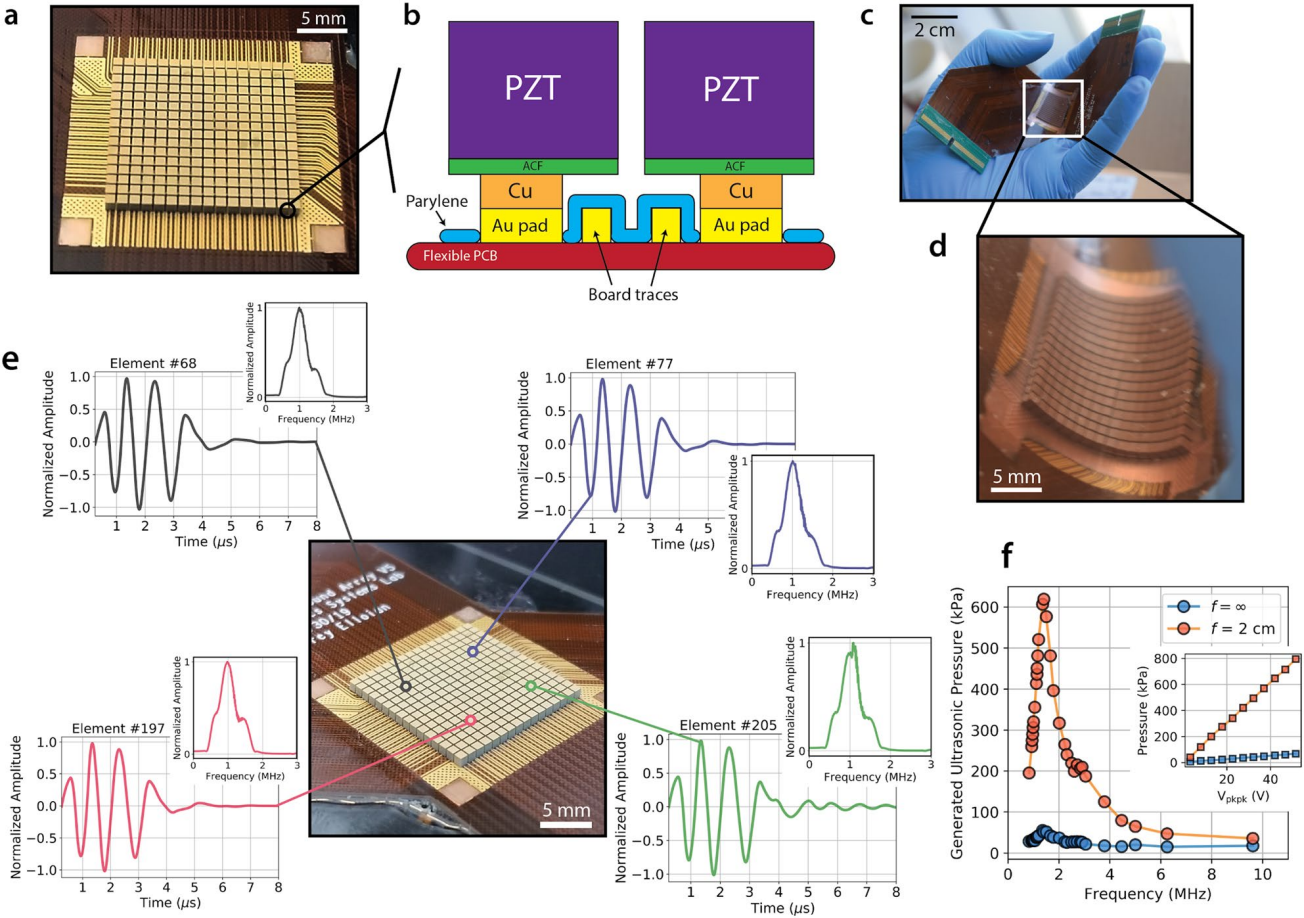
PZT pillar fabrication and ultrasound performance benchmarks of the FlexArray. (**a**) Photograph of the bonded PZT array quarters after back-side dicing to separate out the individual transducer pillars. (**b**) Cross-sectional illustration (not to scale) of the finalised pillar mount. Cu electroplating is used to raise the metal connection before bonding with the bulk PZT transducer on the ACF substrate. Parylene isolates the signal pads from the electrical traces on the board. (**c**) Photograph of the completed FlexArray, relative to a human hand. (**d**) Close-up photograph of a bent piezoelectric array in the center of the device after metallisation and encapsulation. (**e**) Examples of pulse-echo responses, and their associated frequency spectra as insets, from arbitrary pillars in each quarter of the array. The temporal envelope for three pulses is ~ 4 μs with a wide bandwidth of ~ 41%. (**f**) Ultrasonic pressure generated by the FlexArray in water when focused at *f* = *∞* (blue) and at *f* = 2 cm (red), across a range of ultrasound frequencies. The pressure value peaks at over 600 kPa. The inset shows the linear scaling of the generated ultrasonic pressure with applied peak-to-peak voltage across the transducer elements.

Figure 2a shows the diced PZT blocks after the bonding step is carried out to mount them on the electrical pads in the centre of the flexible PCB. A cross-sectional diagram of two neighbouring transducer pillars is shown in Fig. 2b. The transducers are electrically separated from each other, and from the conductive traces on the PCB, with an insulating layer of parylene. In order to minimise potential electrical cross-talk, the signal pads are made taller than the height of the board traces with an intermediate electroplating step before bonding. Once the FlexArray is fabricated, it retains all of the flexibility and ease of handling of the bare PCB, as shown in the photographs in Fig. 2c, d.

We benchmark the ultrasound transduction performance of the finished device by first considering the impulse response of the array in a neutral (unflexed) position. Figure 2e presents four experimental pulse-echo traces, each from a randomly chosen transducer in a different quarter of the 16 × 16 pillar array, and their associated frequency spectra. As evident from the nearly-identical plots, the element-to-element variation in the performance across the array is negligible (with average peak-to-peak voltage variations of < 1.7% and temporal variation of < 1.0%), confirming the reliability of the fabrication process and ensuring consistent device functionality regardless of bending direction. The flexible PCB under the PZT pillar is composed of copper and polyimide with a total thickness of 230 μm. The ultrasonic pressure generated by the array, when tested in water, is ~ 1.4 kPa V^−1^ (~ 15.2 kPa V^−1^ at focus), which is comparable with other flexible ultrasound platforms^19–21^. These response parameters meet the general requirements for high-frequency ultrasound biomedical imaging (1–15 MHz, 20 kPa V^−1^)^22,23^. The SNR, as estimated using the standard expression:

$$SNR = 20log \left( {\frac{{V_{signal} }}{{V_{noise} }}} \right)$$

is calculated to be ~ 49 dB. A frequency sweep (Fig. 2f) for the array, both when unfocused and focused, reveals the resonant frequency of the device to be ~ 1.4 MHz, and a small anti-resonance peak is seen emerging at ~ 2.7 MHz. The response echo bandwidth for single-pulse excitation is 41.3%, which is comparable with recent flexible array technologies^11^, but still trails behind rigid 2D commercial probes, whose bandwidth is often in excess of 70% at low frequencies. Future designs that include flexible matching layers to engineer the acoustic impedance may improve this response. In the inset, we plot the generated ultrasonic pressure, at resonance, as a function of the voltage applied across the array transducers, demonstrating the expected linear scaling at focal lengths (*f*) of both *f* = ∞ and *f* = 2 cm.

### Ultrasound beam steering

Each transducer on the device can be biased with a voltage of an arbitrary phase. The relative offset of these individual phase delays creates patterns of constructive/destructive interference. This allows the FlexArray to steer the ultrasound beam in specific directions in three dimensions at an arbitrary focal point. Such phase steering implementations can have multiple applications, including highly concentrating a pressure beam at a specific location for increased imaging contrast.

**Figure 3:**
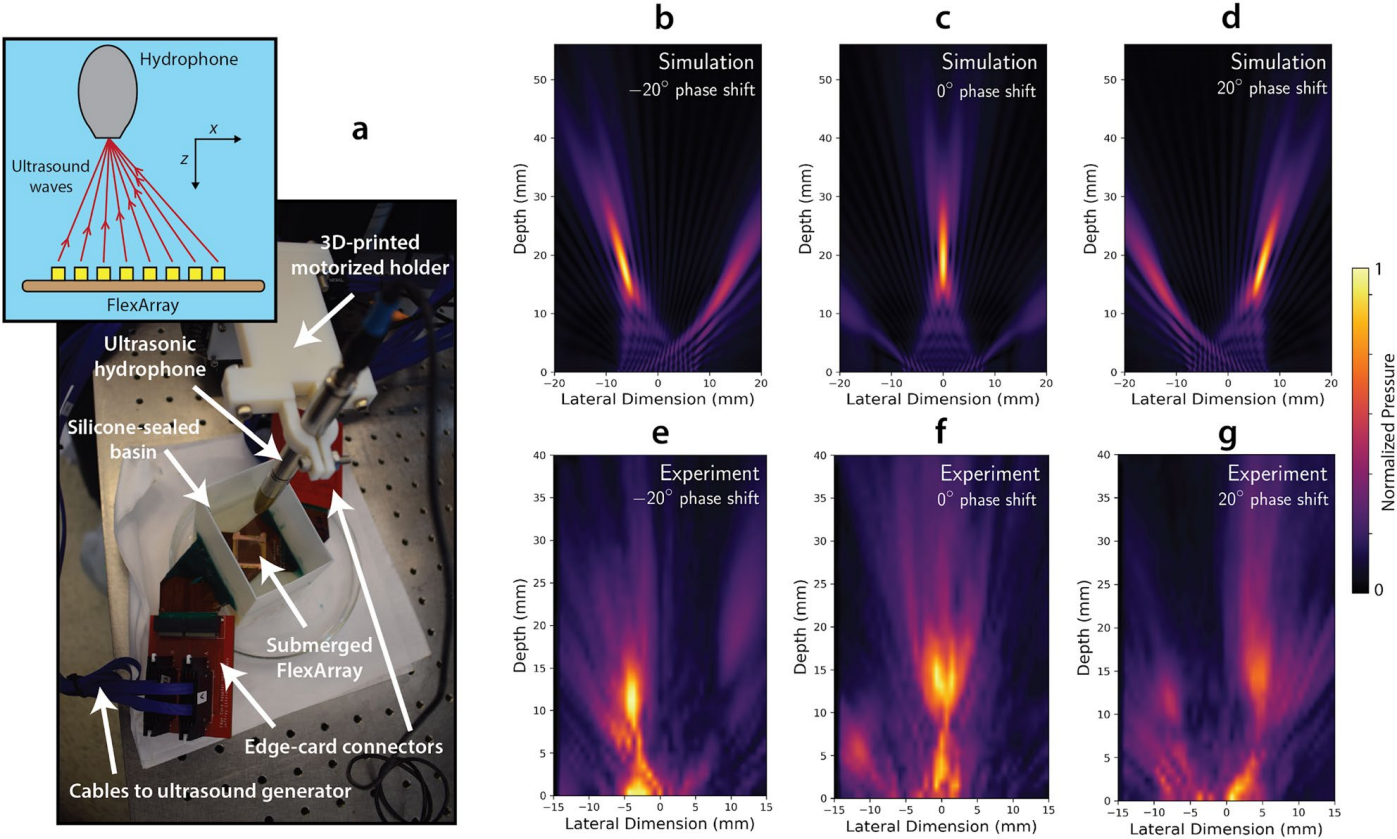
Phase steering of ultrasound beams with the FlexArray. (**a**, Photograph of the experimental setup used to record the distribution of pressure in a water tank at a focal length of 2 cm. Inset: illustration of the detector geometry in the water tank (not to scale). (**b–d**) Simulated pressure maps at this focal length when the array elements are excited at − 20°, 0° and 20° of phase shift, respectively. (**e–g**) Experimental pressure maps collected with the FlexArray at the same respective phase shift values. Note that all colour maps are self-normalised to the colour bar on the right of the figure.

To demonstrate ultrasound beam steering functionality with the FlexArray, we used a custom configuration script to excite the array elements in order to produce a deflected ultrasonic pressure beam in water, in both the *x–z* and *y–z* planes, and we measured the pressure at each location in the *z*-direction, using a hydrophone on a motorised stage. The FlexArray was mounted to a custom 3D-printed part, designed to hold the device within a 50 mm × 70 mm × 60 mm water tank, as shown in Fig. 3a. Simulated beam patterns were produced using the FOCUS II MATLAB package^24^, to predict the behaviour of the device when focused at 2 cm and steered at − 20°, 0, and + 20° of phase difference along the *x–z* plane. The resulting simulated beam patterns for those respective phase shifts are shown in Fig. 3b–d, demonstrating a clear concentration of ultrasound pressure to narrow areas of the array’s field of view. The measured experimental results from the FlexArray for the same respective phase shifts and focus are shown in Fig. 3e–g. The experimentally realised ultrasound beam pattern shows a good qualitative match with the patterns predicted by simulations, demonstrating spatial ultrasound beam steering capability as well as axial pressure hotspot localisation. The qualitative mismatch originates from near-field interference effects near the surface of the experimental device that causes additional hotspots to emerge at lower *z* values. As the device is designed to operate at low frequency for deeper tissue penetration, these artefacts do not play a big role in image formation beyond 2 cm of penetration depth. The maximum value of focal pressure achieved with the FlexArray is ~ 615 kPa, at a voltage of ± 20 V at a focal length of 2 cm. Improvements to ultrasound coupling to biological media with the potential inclusion of a matching layer on the surface of the PZT pillars may enable even higher sensitivity in the future. For more simulations and experimental results when the array-generated beams are focused at infinity, see Supplementary sections 4 and 5.

### Transducer cross-talk characterisation with increasing curvature

**Figure 4:**
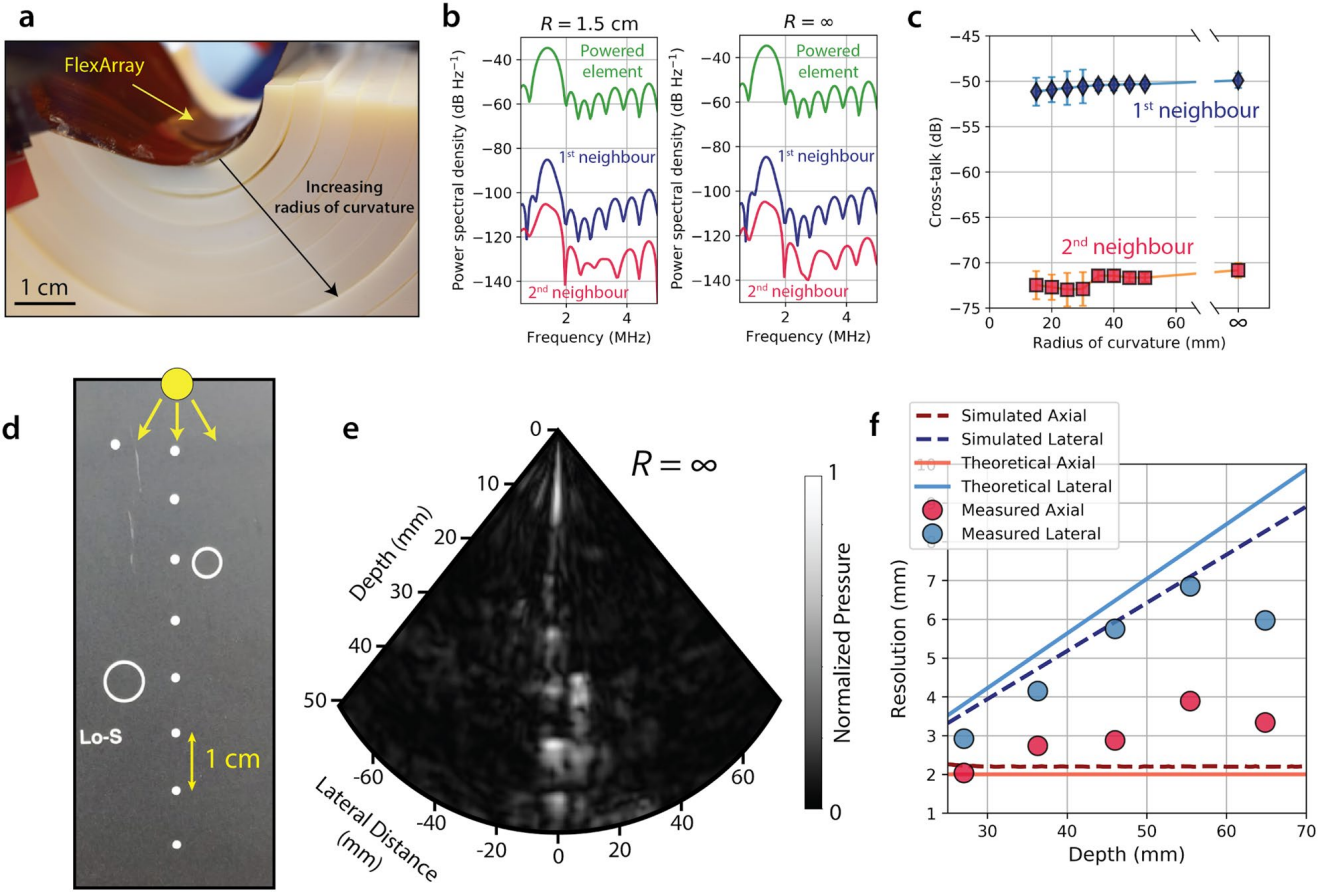
Cross-talk characterisation between neighbouring elements and medical-grade flat phantom imaging. (**a**) Photograph of the detachable 3D-printed apparatus for the testing of electrical cross-talk across different radii of curvature. (**b**) Power spectra of the voltage response of a single powered transducer (green), relative to those of its nearest neighbour element (blue) and its second nearest neighbour (pink). Extreme cases of a highly-curved array (*R* = 1.5 cm) and a flat array (*R* = ∞) are compared. (**c**) Cross-talk between neighbouring elements, extracted at 1.4 MHz, as a function of radius of curvature of the FlexArray. The device shows excellent inter-channel shielding characteristics at high operational frequencies, regardless of shape. (**d**) Photograph of the cross-section of the tissue phantom with embedded nylon wires (white dots). The yellow dot marks the vertical location where the FlexArray was placed during imaging. (**e**) B-mode scan of the phantom taken with the FlexArray, demonstrating both its axial and lateral resolution. The locations of the nylon rods can be resolved axially at ~ 1 cm apart. (**f**) Plot of the theoretical, simulated, and measured axial/lateral resolutions as a function of scatterer depth. Note that error bars are smaller than the marker size of the experimental data points. In both simulations and experiments, the number of *x–z* raylines is 96.

To ensure that the PZT pillars are electromechanically isolated at all instances of bending, we examined the inter-pillar electrical cross-talk on the array as a function of the radius of curvature, *R*. Figure 4a shows a photograph of a set of detachable 3D-printed models which allow for bending the FlexArray to a specific radius of curvature between 1 and 5 cm. We characterised the electrical cross-talk between an individual powered element and its nearest two neighbours across this radius of curvature range based on their power spectral densities (extreme cases of *R* = 1.5 cm and *R* = ∞ are presented in Fig. 4b). In Fig. 4c, we plot the relative cross-talk power between the powered-on element and its first and second neighbours, as a function of *R*. Each radius was tested 10 times on the same set of elements, and the error bars correspond to one standard deviation from the mean. The average recorded cross-talk magnitudes are ~ − 50 dB for the nearest neighbour element and drop to under − 70 dB when measured two elements away. Importantly, the cross-talk levels remain unchanged as the array is flexed away from the neutral position all the way to extreme radii of curvature below 2 cm. This particular array was mechanically cycled > 100 times throughout this testing in the curved molds to generate these data points and did not incur any noticeable change in electrical performance when tested again in the neutral position. In addition, there was no visible elastic deformation to the PCB, highlighting the robustness of the packaging. Although piezoelectric composites may lead to higher cross-talk suppression than PZT due to their anisotropic phonon dispersions^25–27^, they offer the potential for only a small SNR increase of ~ 1.1 dB and additional cross-talk suppression of only ~ 10 dB to the nearest element over the PZT elements employed here^11^.

### B-mode imaging of test phantoms

After confirming the electrical functionality of the FlexArray, we first imaged the medical-grade *84-317* Multi-Purpose Tissue/Cyst Ultrasound Phantom (*Fluke Biomedical*) with the FlexArray in a flat position (*R* = ∞). The composition of the *84-317* phantom is designed to mimic the acoustic properties of the human liver, with additional buried ultrasound reflectors consisting of 0.24-mm diameter monofilament nylon rods. To demonstrate the resolution of the array, we imaged a set of coplanar parallel rods located 1 cm apart in the axial direction (see photograph in Fig. 4d). A composite image was created by weighted averaging of scans focused at depths of 4 cm, 5 cm, 6 cm, and 7 cm. This resultant B-mode image (Fig. 4e) matches well with the known locations of the reflectors in the phantom. Additional scatterers from an adjacent set of rod targets are also seen in the photograph; however, the reflected intensity in the B-mode from the low scatter cyst (Lo-S) region remains unresolved, as the core is designed to be more transparent to ultrasound. We extracted the axial and lateral resolutions (see definitions in Methods) as a function of imaging depth from the phantom images (circular data points in Fig. 4f). We performed a series of simulations in FIELD II utilising the same array geometry at 1.4 MHz, with a single point reflector located at arbitrary points along the *z* axis, and plotted the resulting resolutions (dashed lines) along with the theoretical expected resolutions (solid lines). The experimental data points show a good trend correlation with the measured point targets in the phantom, expectedly trailing off at larger depths due to increased scattering in the medium. Future improvements to the image resolution achieved here will come from migrating our fabrication process to CMOS-based electronics. By reducing the footprint of the whole array, increasing the transducer and interconnect density and thinning the piezoelectrics for higher frequency operation, future devices based on this integration paradigm will be able to carry out real-time B-mode imaging with sub-mm resolution. For a direct comparison of the key parameters of this study with other similar ultrasound imaging arrays in the recent literature, see Table S1.

We developed a custom geometric phasing algorithm to apply imaging corrections based on a known radius of curvature in the *x* and *y* axes. More details of this corrective algorithm are presented in Methods. We also provide an additional discussion in the Supplementary Information on the potential applications of time-of-arrival algorithms for automatically detecting the radius of curvature of the array by pulsing individual transducers. We discuss the challenges associated with the sensitivity of the extracted radius of curvature to the error in the measured transducer positions in Fig. S11. For simple cylindrical bending around an axis, the corrected coordinates can be computed from their respective original coordinates using the transformations in Table 1. In the FIELD II simulations used here^28^, different radii of curvature are applied to the transducer array and the image of a rectangular 10 mm × 5 mm air pocket suspended in gelatin at a distance of 2 cm from the imaging aperture is reconstructed^29^. In order to accurately bend the array to a known *R* for experimental imaging of the air pocket, custom moulds were 3D-printed as gelatin casts. These exchangeable shapes could then easily be used to cap the air pocket with a curved piece of a known *R* for each measurement.

**Table 1:** Adjusted phasing coordinates transformed according to the corresponding radii of curvature in the *x–z* and/or *y–z* planes.

	Curve in *x–z*	Curve in *y–z*	Curve in *x–z* & *y–z*
*x*_*0*_	$R_{x} sin\left( {\frac{x}{{R_{x} }}} \right)$	$x$	$R_{x} sin\left( {\frac{x}{{R_{x} }}} \right)$
*y*_*0*_	$y$	$R_{y} sin\left( {\frac{y}{{R_{y} }}} \right)$	$R_{y} sin\left( {\frac{y}{{R_{y} }}} \right)$
*z*_*0*_	$$R_{x} \left( {1 - cos\left( {\frac{x}{{R_{x} }}} \right)} \right)$$	$$R_{y} \left( {1 - cos\left( {\frac{x}{{R_{y} }}} \right)} \right)$$	$$R_{x} \left( {1 - cos\left( {\frac{x}{{R_{x} }}} \right)} \right) +$$$$R_{x} \left( {1 - cos\left( {\frac{y}{{R_{y} }}} \right)} \right)$$
Azimuth	$tan\left( {\frac{x}{{R_{x} }}} \right)$	$0$	$tan\left( {\frac{x}{{R_{x} }}} \right)$
Elevation	$0$	$tan\left( {\frac{y}{{R_{y} }}} \right)$	$tan\left( {\frac{y}{{R_{y} }}} \right)$

**Figure 5:**
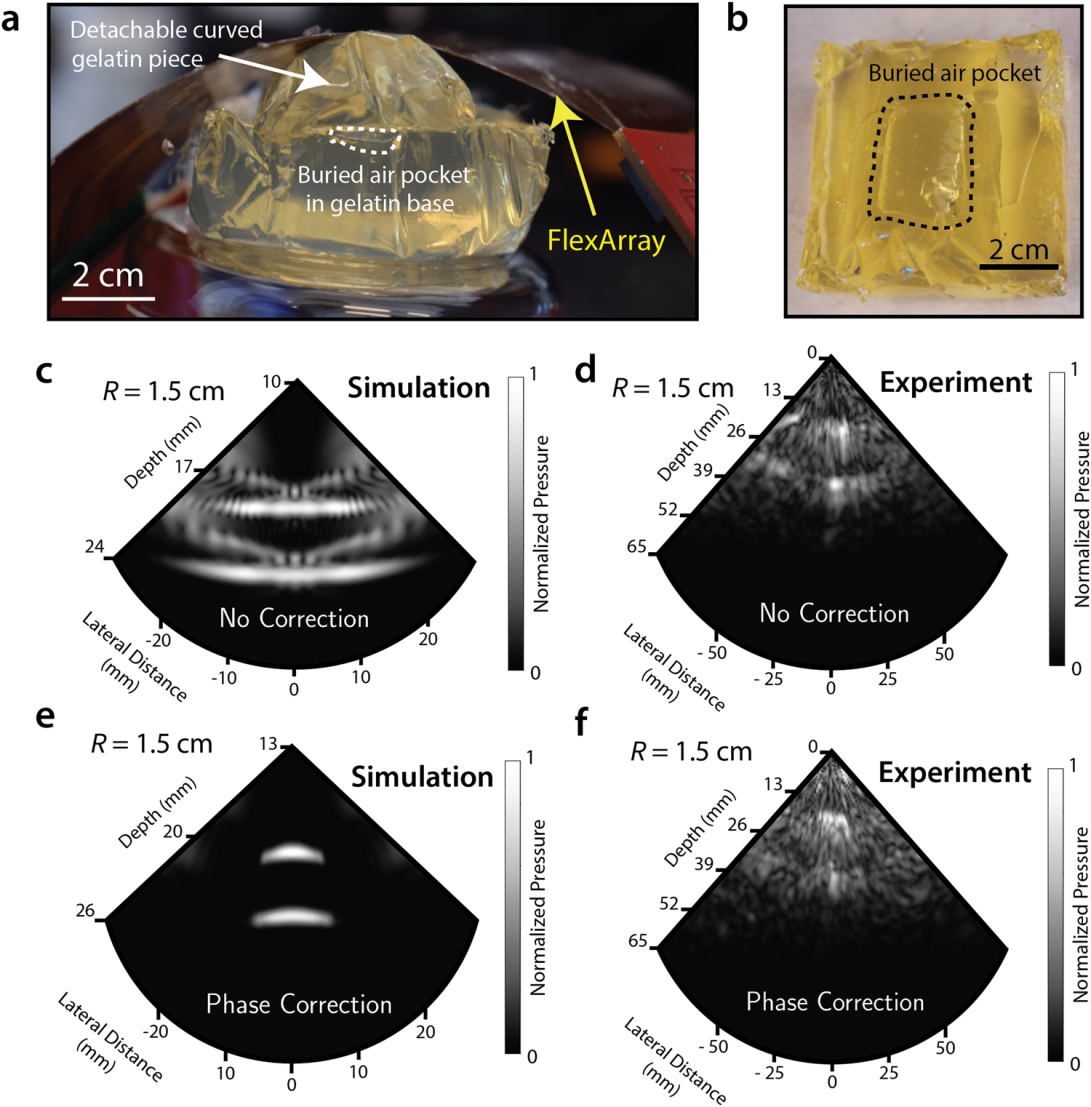
Effects of phase correction on image formation tested on gelatin phantoms of arbitrary curvature. (**a**) Photograph of the cast gelatin phantom used to collect B-mode images at arbitrary curvatures. The FlexArray is seen conforming to the surface. (**b**) Plan-view photograph of the air pocket-containing gelatin mould. (**c**) Simulated B-mode image when the radius of curvature is 1.5 cm and phase correction is not implemented. (**d**) Experimental B-mode image of an air pocket in gelatin captured with the FlexArray when the radius of curvature is 1.5 cm; without phase correction. (**e**) Simulated B-mode image when the radius of curvature is 1.5 cm and phase correction is implemented. (**f**) Experimental B-mode image of an air pocket in gelatin captured with the FlexArray when the radius of curvature is 1.5 cm; with implemented phase correction. Both images in (**d**) and (**f**) are compound averaged focal series images between *f* = 2, 3 and 4 cm.

For the case of *R* = 1.5 cm, we compare simulations with the experimental performance of the FlexArray—with and without geometric phasing correction. Figure 5a, b present labeled photographs of the experimental set-up from the side and top views, respectively, highlighting the location of the air pocket. Figure 5c shows the simulated B-mode scan without phase correction. The two main high-intensity features seen in the plot are regions of high reflectivity from the top and bottom of the air pocket.

When using our phase delay correction in simulation (Fig. 5e), the edges of the air pocket become clearly defined in the B-mode image, confirming the functionality of the correction algorithm. For simulated plots at different *R* values, see Supplementary section 4. Figure 5d, f present the same B-mode comparison, but for experimental data obtained with the FlexArray, without and with phase correction, respectively. As in the simulation in the absence of phase correction, spurious reflections around the air-gelatin interfaces increase the perceived lateral width of the air pocket. When the phasing algorithm is implemented, the air-gelatin interface is correctly resolved. The extent of the strong reflection is also narrowed in the axial direction for the two interfaces. We note that the experimental sonograms are compound images of B-modes taken at three different focal lengths (*f* = 2, 3, 4 cm), appropriately weighted with windowing functions to remove the high-intensity speckle region from the near field. We remark here that the B-mode data are presented in grayscale to match the typical experience of an ultrasound operator. In addition, we also present the same data plotted with a divergent colormap in Fig. S12, where areas of high-pressure may be more easily identified.

For image formation, we utilized the flash echo technique in TX mode, and traditional delay-and-sum with phase correction on the RX side. This allows us to achieve a real-time frame rate of 4.5 frames/s in the current prototype, but this could also be improved further with more efficient programming paradigms and processing hardware in the future.

### In-vivo imaging of the human humerus

**Figure 6:**
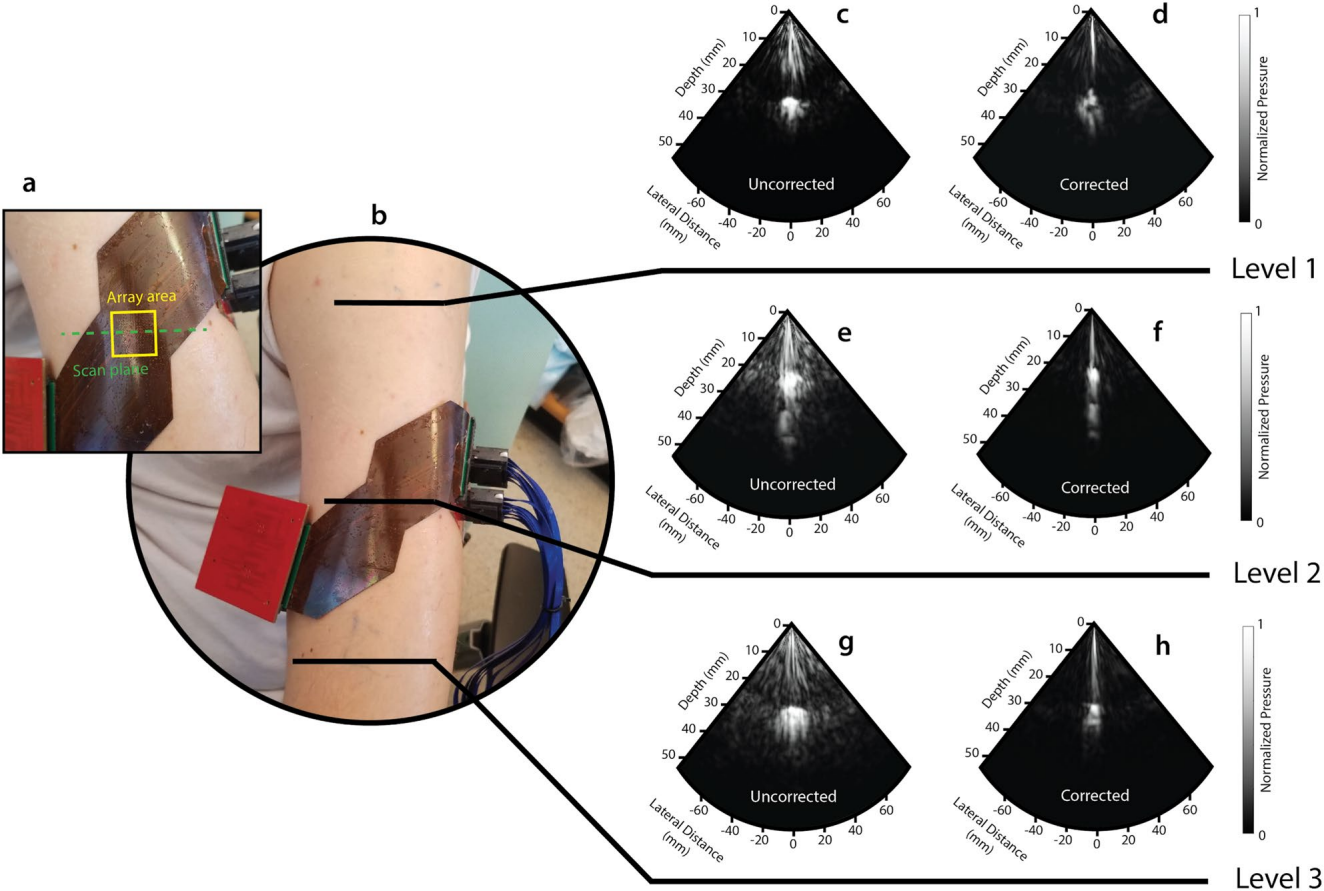
Imaging of the human humerus with the FlexArray. (**a**) Close-up photograph of the array area on the arm during imaging, also showing the 2D plane along which the scan was taken. (**b**) Photograph of the subject’s arm with the device in the Level 2 position. Three levels were chosen to image the bone along the length of the arm. (**c**, **d**) Level 1 B-mode images of the humerus without and with correction for the radius of curvature of 4 cm, respectively. Analogous images were taken at (**e**, **f**) Level 2 and (**g**, **h**) Level 3.

The system was also tested with curvature in vivo in humans by wrapping the device directly around the arm of a healthy human male. To demonstrate the effectiveness of the system on a curved body part, we imaged the humerus bone at three different levels along the arm, with the scan plane orthogonal to the length of the arm (Fig. 6a, b). Details of the experimental procedure can be found in Methods. The exact radius of curvature to be corrected for is pre-determined by measuring the *R* in different positions on the arm of the subject with 3D-printed molds that have a set *R* value. We note here that several other approaches to accurately determine R are being pursued in the literature, namely echo response-based algorithms^30^, deep learning methods^31^ and sensor-mediated approaches^32^. It remains to be seen whether software-based methods are accurate for the varied clinical cases and different array designs, while introducing sensors such as extensometers increases the device bulkiness and complicates packaging. Our static correction ensures the phasing algorithm is applied correctly.

We note that high-reflection artefacts are present in the first 1 cm of each scan, due to near-field interference effects, but the device is not designed to image any structures at such shallow depths given its low frequency of operation, so these features can be neglected. We imaged three different regions of the humerus bone: the surgical neck (Level 1 in Fig. 6), the deltoid tuberosity (Level 2 in Fig. 6) and the shaft (Level 3 in Fig. 6). These all have a slightly different bone radius and bone surface location relative to the top of the skin and as such, are expected to produce distinct features in the sonogram. The B-mode images in Fig. 6c, e, g were taken without any phase correction at each level, whereas those in Fig. 6d, f, h were taken at the same corresponding levels, but after applying a correction for a radius of curvature of 4 cm in both the *x–z* and *y–z* dimensions. The phase correction algorithm allows the central feature to be more clearly defined, and removes spurious signals seen at the peripheral scan angles. In both uncorrected and corrected images, the predominant feature is the surface of the humerus where a fraction of the top part of the bone, approximately 2 cm in diameter, can be seen. When phase correction is applied, this reflection narrows laterally by more than 30% in all three locations. Assuming a circular cross-section for the bone, the imaged bone surface corresponds to a bone diameter of ~ 2 cm, falling within the expected range for an adult human male^6,7^.

We remark here that visualising bone is a rather basic benchmark in terms of in-vivo imaging, due to the relatively high reflectivity and shallow tissue depth. With commercially-available rigid probes, the operator can usually apply higher pressure with the device to achieve better sensitivity when imaging these features. The advantage of a 2D flexible probe is the ability to correct for imaging artefacts which—with improved resolution in future designs—may help to identify sub-mm features during emergency procedures without requiring advanced skills from the operator. The FlexArray images match well with the expected bone locations, as evident from comparative B-mode scans taken with a commercial *Philips* P4-1 probe on the same human subject (see Fig. S14). In addition, Fig. S15a, c, e and S15b, d, f show 3D representations of 16 *y–z* slices, without and with the corrective algorithm. Similar to the case of 2D images, the phase correction removes spurious false reflections seen in the uncorrected images along the length of the humerus. A phase-corrected B-mode scan video of the Level 1 location of the humerus, as the subject’s triceps muscle is flexed and relaxed in real time, is also supplied in Supplementary Movie 1.

## Discussion

In this work, we have demonstrated a prototype passive flexible ultrasound phased piezoelectric array with curvature correction. The geometric phasing algorithm allows for improvements to image quality by removing artefacts when visualising the internal structure of soft matter on curved surfaces to which the array can conform—including those of the human body. Clinical applications require further improvements in imaging quality which will result from integrating more elements in the imaging array. This can only be achieved with tighter integration of the transceiver electronics than is demonstrated in the current passive transducer array design here. In particular, to push the technology into a fully wearable ultrasound imaging device, transducers should be integrated directly onto complementary metal–oxide–semiconductor (CMOS) substrates containing all the transceiver electronics^33^. When thinned, these substrates can also be rendered flexible. Current prototypes of wearable ultrasound arrays demonstrated in the literature—as well as the one presented here—all rely on interfacing the array with bulky electronics, i.e., the multi-channel data acquisition hardware, edge card adapters, wiring, the flexible PCB and the *Verasonics* signal generator itself. Direct integration on CMOS will allow for a reduction in transducer pitch, the integration of more elements in the array, and the integration of all the control electronics onto the wearable device, resulting in a truly tether-less form factor. The low-temperature process of transducer integration developed here is directly translatable to these flexible CMOS substrates. In addition, further work on fabricating a matching layer on top of the bulk transducer sheet *before* dicing and integration would also increase the acoustic pressure coupling, bettering the sensitivity range of the device.

## Methods

### Device design, fabrication and packaging

The process workflow for fabricating the FlexArray is described in detail in section 1 of the Supplementary Material. The scanning electron micrographs of the whole pillar array were taken before parylene encapsulation at a beam energy of 20 keV, while the high-magnification images of the PZT surface were taken at 4 keV. For energy-dispersive X-ray spectroscopy spectra collection, the stage was tilted at 15° towards the detector, using a 30 μm aperture at 20 keV.

### Simulations and geometric phasing algorithm

In the case of a simple linear array of radiating elements, the time required for a pressure front to arrive from a given element to a target point, *P(r, θ)*, is dependent on the distance between the point and the element, as well as the wave velocity in the medium. In a simple 1D case, we can describe the distance of the *n*-th element (*r*_*n*_) to a point with Eq. (1):

1 $$r_{n} = \sqrt {r^{2} + x_{n}^{2} - 2rx_{n} sin\left( \theta \right)}$$

The near-field Fresnel approximation can then be used to simplify the expression for the relative positional difference for the *n*-th element^34^, in the limit where *r* >> *x*_*n*_:

2 $$\Delta r_{n} = r - r_{n} = r - \sqrt {r^{2} + x_{n}^{2} - 2rx_{n} sin\left( \theta \right)} \approx x_{n} sin\left( \theta \right) - \frac{{x_{n}^{2} }}{2r}$$

Provided that the longitudinal speed of sound, *c*, is known in the propagation medium, the relative time delay required to cause constructive interference at the desired focus is:

3 $$\Delta t_{n} = \frac{{\Delta r_{n} }}{c} = \frac{{x_{n} sin\left( \theta \right)}}{c} - \frac{{x_{n}^{2} }}{2cr}$$

In this form, the first term determines the delay due to the steering angle, whereas the second term is the contribution from the focus. It is therefore possible to only apply a steering angle to generate a plane wave in a desired direction. By staggering the delays between elements, the pressure beam can be steered with or without a focus.

For the case of a 2D array, Δr_n_ becomes a 3D vector. When curving the array, the initial centroids of the elements, as given by *x*, *y*, and *z*, must be geometrically transformed by the radius of curvature in the *x*–*z* plane, *y*–*z* plane, or both, depending on the shape of the bent device. The correct phase delays may then be calculated using the transformed coordinates, *x*_*0*_, *y*_*0*_, and *z*_*0*_, as given in Table 1. The beam steering simulations were performed in the FIELD II MATLAB package to confirm the proper reconstruction of a block of air suspended in gelatin, situated parallel to the aperture, and 2 cm away^29^. In simulation, the block of air was represented by tightly-packed (100 μm-pitch, or ≈ 0.2 λ) perfect reflectors, where λ is the ultrasound wavelength and the excitation was a single sinusoidal pulse at 2 MHz.

The COMSOL 5.6 Multiphysics simulations were carried out using a 3D pillar stack-up geometry, with material parameters from the COMSOL library, utilising the Solid Mechanics, Electrostatics, Pressure Acoustics (Frequency) and Multiphysics modules with periodic boundary conditions on the sides of the transducer. The ultrasound response was probed in water, a few millimeters above the pillar, in the frequency domain between 1 and 3 MHz.

### Ultrasound and electromechanical testing of the array

The array was first tested on a flat surface, mounted within a 3D-printed (*Stratasys Connex3 Objet260*), water-tight testing apparatus. These initial tests confirmed the operation of the array by producing beam patterns. Beam steering and focusing were independently controlled by means of setting the delays on the graphical user interface of the MATLAB software controlling the *Verasonics Vantage* system. An *Onda HGL-0200* hydrophone was used in conjunction with a motorised stage to sample the pressure in a grid along the *x–z* plane, with a grid resolution of 1 mm.

For the B-mode imaging of the curved gelatin phantoms, 85 g of uncoloured gelatin (*Knox)* were dissolved in 500 mL of boiling water before pouring this mixture into 3D-printed moulds of a pre-defined radius of curvature (in a range of *R* = 1–3 cm). After curing the moulds at 4 °C for 3 h, the gelatin phantoms were removed for testing. One set of moulds was always used as the bottom part—it was cast as a flat 5 cm by 5 cm by 2 cm block, with an air cavity located at the top center, with dimensions of 2 cm by 1 cm by 0.5 cm. In this way, the top half of the gelatin phantom could be easily changed out between measurements and offered an exact radius of curvature, while always measuring the same air pocket target. The FlexArray was manually wrapped around the curvature of the gelatin phantom to record each B-mode image.

### Defining the resolution of the FlexArray on a medical-grade phantom

We defined the resolution in the lateral and axial dimensions by finding the half-power beamwidth (− 3 dB) of the corresponding reflected echo along the horizontal and vertical axes, respectively. The theoretical axial resolution should be constant with depth, and depends on the quality factor, *Q*, wavelength of ultrasound, *λ*, and the number of pulses, *n*:

4 $$AR_{theo} = \frac{Q\lambda n}{4}$$

Similarly, given a depth, *z*, and array aperture, *L*, the theoretical lateral resolution as a function of depth can be given by:

5 $$LR_{theo} = \frac{2\lambda z}{L}$$

Experimentally, the B-mode was taken on the top surface of the *84-317* Multi-Purpose Tissue/Cyst Ultrasound Phantom (*Fluke Biomedical*), with the array lying flat (*R* = ∞), centered above the column of buried nylon strings, each separated axially by 1 cm. The transducer excitation voltage was ± 10 V, with 2 pulses used to form the image.

### In-vivo imaging of the human humerus

Approval was obtained from the Columbia University Institutional Review Board to carry out ultrasound imaging experiments on volunteer human subjects (IRB No. AAAS6542). All methods were carried out in accordance with relevant guidelines and regulations. Informed consent was obtained from the subject before testing. During imaging, the subject was conscious for the entirety of the procedure and was able to provide feedback in real time. The FlexArray was placed directly on the skin at various locations on the arm, and standard ultrasound coupling gel was used (*Aquasonic 100*). The subject’s arm remained stationary as the scans were taken. The radius of curvature of the arm was measured at three different points, by matching to a 3D-printed mould of a fitting size. This *R* was then used for image correction during the scan. Images were taken with an applied voltage of ± 10 V to the transducers, which corresponds to a peak pressure of less than 200 kPa at the focal point.

## Supplementary Information

Supplementary Video 1.

Supplementary Information 1.

Supplementary Information 2.

## Data Availability

Data collected in this study are available from the corresponding author upon reasonable request.
