# Supplementary material for: Flexible ultrasound transceiver array for non-invasive surface-conformable imaging enabled by geometric phase correction
Source: Sci Rep. 2022 Sep 28;12:16184. doi: 10.1038/s41598-022-20721-7 (PMC9519534; doi:10.1038/s41598-022-20721-7)
Supplement: Supplementary file 2 — Supplementary Information 1. [file 41598_2022_20721_MOESM2_ESM.docx]

**Supplementary Information:**

**Flexible ultrasound transceiver array for non-invasive surface-conformable imaging with geometric phase correction**

*Jeffrey Elloian,^1, *^ Jakub Jadwiszczak,^1, *^ Volkan Arslan,^1^ Jeffrey D. Sherman,^1^ David O. Kessler^3^ and Kenneth L. Shepard^1,2^*

**Affiliations**

^1^Department of Electrical Engineering, Columbia University, 500 W 120th St., New York, New York 10027, United States.

^2^Department of Biomedical Engineering, Columbia University, 1210 Amsterdam Avenue, New York, New York 10027, United States.

^3^Department of Emergency Medicine, Morgan Stanley Children’s Hospital of New York Presbyterian at Columbia University Medical Center, New York 10032, United States.

*These authors contributed equally.

**Supplementary Section 1. Piezoelectric transducer array design and fabrication**

The overall design flow to fabricate the FlexArray begins by electrically contacting a sheet of PZT piezoelectric material by patterning pads on either side. The flexible printed circuit board (PCB) is prepared for transducer integration by building up the pad heights through electroplating and creating isolating parylene pockets. Subsequently, pillars are then formed in the PZT sheet by mechanical dicing and are bonded to the substrate. A sputter metallisation step is used to provide the top ground connection. Finally, the device is encapsulated with parylene to prevent electrical shorts from contact with the environment. We discuss the fabrication flow in detail below and present a sketch of the cross-section of the array at each key step in Fig. S1.


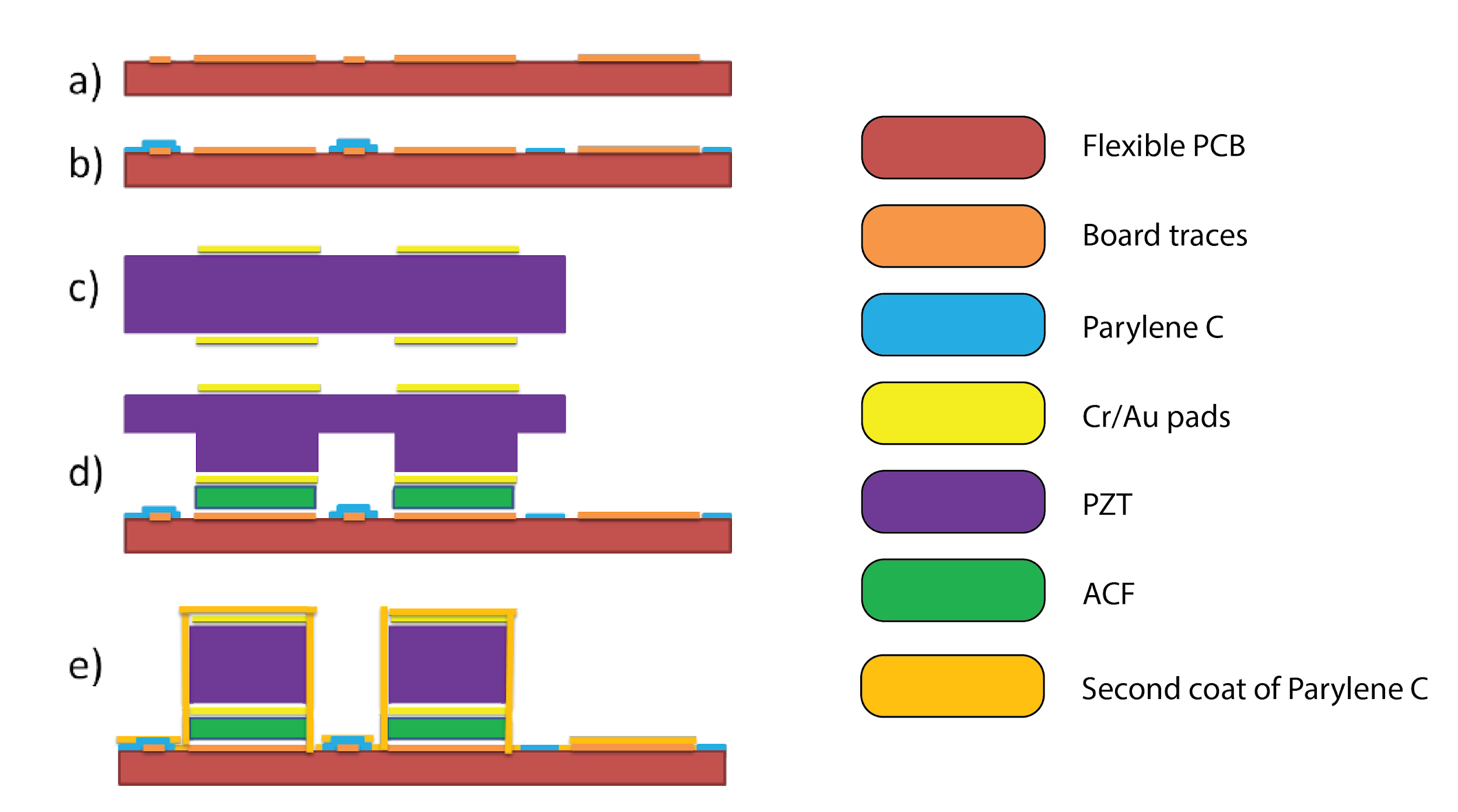


**Fig. S1. Cross-sectional fabrication flow for the FlexArray. a** The as-produced flexible PCB with marked board traces and contact pads. **b** Parylene coating isolating the pads from the traces. **c** PZT block with lithographically defined pads, before bonding to the board. **d** The PZT block is bonded to the pads on the board with an intermediary anisotropic conductive film (ACF) layer. **e** Final parylene coat after dicing, electrically isolating all the individual pillars from one another.

A disk of pre-poled PZT-5H (diameter approx. 75 mm, thickness approx. 1016 μm, *Piezo.com*) was used as the starting material. Utilising a pre-poled piezoceramic sheet is amenable to migrating this fabrication flow to complementary metal-oxide-semiconductor (CMOS) processes, as electrical poling procedures involve large electric fields that will destroy integrated circuits during poling. All processing steps, however, need to be conducted at a temperature less than half of the Curie temperature of PZT (*T_C_ =* 340℃) in order to preserve a meaningful piezoelectric effect^1^. Commercial PZT disks are pre-coated with nickel, which is susceptible to oxidation in air. Before fabrication began, the disks were placed in ferric chloride solution for approximately 1 minute to strip the nickel film from the surface of the PZT. Standard photolithographic techniques were then used to pattern a grid of contact pads on either side. These pads were designed to have the same dimensions as the pads on the board, i.e. 16 × 16 symmetric element arrays, with 425 μm pads, with 1 mm pitch. To optimise the amount of usable material per piezoelectric disk, a 4 × 4 grid of these arrays was defined, although the size of the piezoelectric disk usually limited the yield to only the centre-most 12 arrays. There is a spacing of 1575 μm from edge to edge between the pads of each array. Cross-shaped markers were used both for alignment during lithography and as dicing lanes. The mask is shown in Fig. S2a.

To pattern the pads, the PZT block was first treated in a plasma asher (*Anatech*) with O_2_ plasma at 100 W for 5 minutes to remove organic residue, and to chemically prime the surface for adhesion. S1818 photoresist was spun at 500 rpm for 10 seconds to spread the resist initially, and then at 3000 rpm to produce a layer of approximately 1.5 μm in thickness. The resist was then soft-baked at 110℃ for 60 seconds. The UV light exposure was performed using a *Suss MA6* mask aligner with the aforementioned mask at 150 mJ/cm^2^. The pattern was then developed in AZ 300 MIF developer for 60 seconds. To ensure no resist scum remained in the patterned area, the same plasma asher was used to expose the sample to O_2_ plasma at 100 W for 2 minutes. Subsequent metallisation was carried out in an electron beam evaporator (*Angstrom Engineering EvoVac Multi-Process*), depositing a 5 nm adhesion layer of chromium followed by 100 nm of gold – forming the top pads. An example of a PZT sheet immediately after metal deposition is shown in Fig. S2b.

Rather than immediately performing lift-off, the backside of the PZT was patterned first. The PZT disk is flipped to the yet-unexposed back-side, and the same photolithographic process is repeated. During the alignment stage, however, backside alignment was performed on the *Suss MA6* mask aligner, i.e. the microscope lens was situated below the stage. Backside development was performed in AZ 300 MIF again using the same parameters. We note here that over-development of the already-exposed and metallised side is not a concern as the developer does not react with gold. The same plasma ashing and evaporation steps were repeated on the newly exposed backside. The entire disk was then placed in acetone for lift-off and sonicated for 5 minutes to completely remove the resist. This resulted in a PZT disk containing grids of gold pads on either side, which are aligned with each other across the piezoceramic, yielding approximately 12 grids which can be used to make 12 separate arrays.


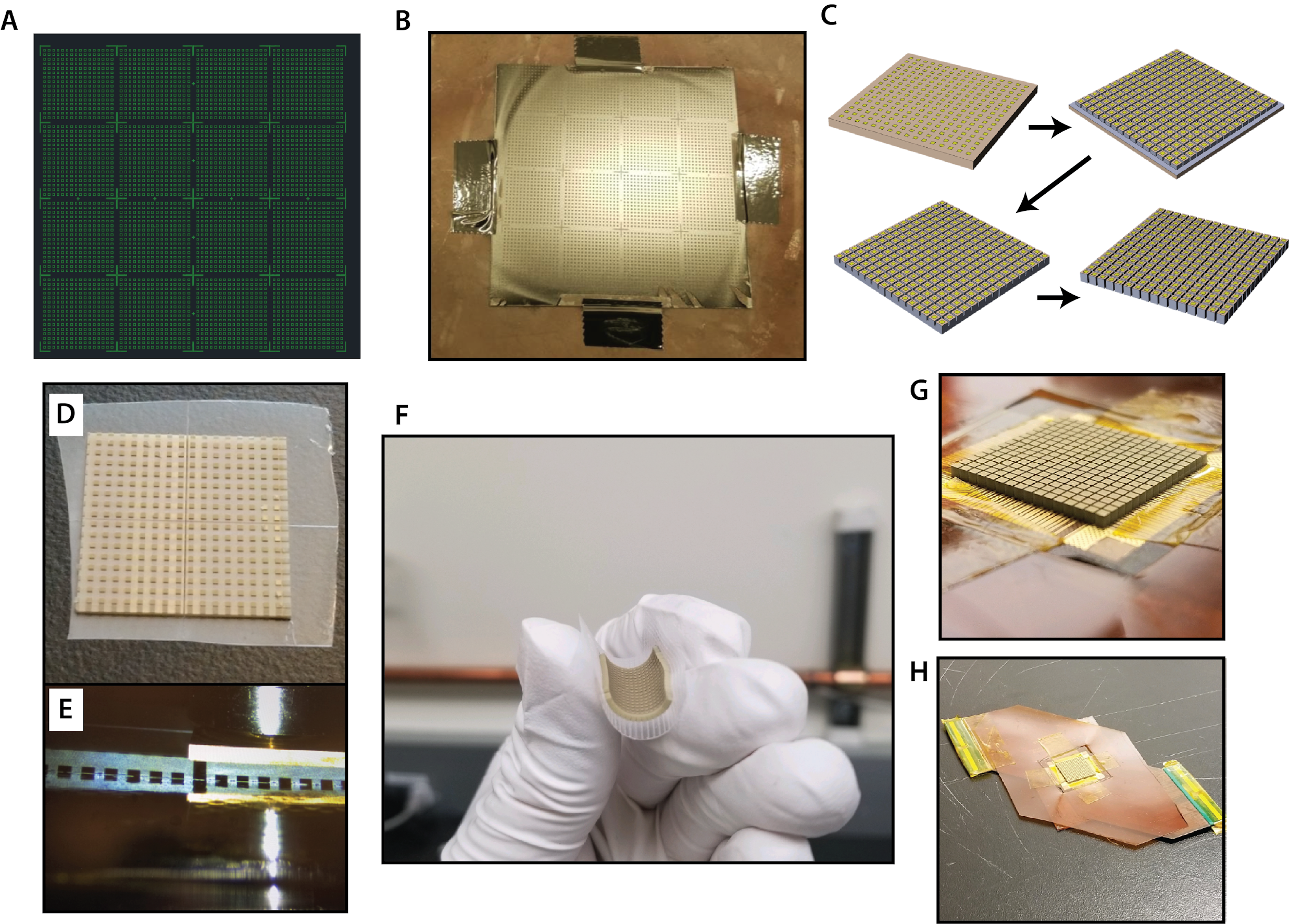


**Fig. S2. Fabrication of the piezoelectric pillar array. a** Computer-aided design pattern of the piezoelectric array. **b** PZT disk after lithographic pattern exposure and metal deposition. **c** 3D rendering of the dicing progression for an individual array. **d** Array quarters after dicing. **e** Camera shot of the array cross-section during the bonding procedure to the flexible PCB. Note the metal toolhead pressing down from the top. **f** Isolated PZT pillars directly mounted to dicing tape to demonstrate flexibility. **g** Photograph of the diced array on the PCB after bonding. **h** Polyimide shadow mask on the board, preparing the array for top metal deposition.

The size of the pads on which the PZT pillars are mounted is limited by the routing of the board traces. These traces between pads also received the same ENIG (two layer metallic coating of 2-8 μin Au over 120-240 μin Ni) surface finish during manufacturing – meaning that on as-produced boards, the pads and the traces both have the same height. This would result in short-circuiting between elements after pillar bonding. Therefore, the pad areas needed to be selectively built-up in order to comfortably protrude above the height of the board traces if their area was large enough to result in this overlap. This was accomplished by electroplating the gold pads and isolating these with parylene to prevent shorts. First, a masking layer was used to only expose the pads as targets for plating. Thick (> 10 μm) AZ P4620 photoresist was spun on at 500 rpm for 5 seconds, followed by 2500 rpm for 45 seconds, and baked at 110℃ for 90 seconds to remove any remaining solvent. Exposure was done with the *Suss MA6* mask aligner, at 300 mJ/cm^2^, followed by development in AZ 400K:DI water (1:4) for 3 minutes. O_2_ plasma was used again to de-scum the sample for 5 minutes at 100 W. A wafer electroplating set-up (*Yamamoto-MS*) utilising cupric sulfate solution was used to build up a copper layer on top of the gold pads. The pads were temporarily shorted with edge card connector pins using acetone-soluble silver paint solution (*TedPella*) such that they were at the same electrical potential and attracted an equal deposition rate for the copper. The whole board was masked in Kapton tape to prevent plating in unwanted areas. The desired exposed area for plating was 0.7164 cm^2^. Given a target current density of 20 mA/cm^2^ to ensure a constant deposition rate, the current was limited to 14.33 mA, which required a 140 mV bias. The throw distance between the copper anode and the target PCB was fixed to 4.5 cm. The deposition rate was approximately 6.9 nm/sec for a target height of 12.45 μm after 30 minutes of plating. After plating, the entire board was placed in acetone for 10 minutes to remove both the resist mask, as well as the silver paint.

To ensure the board was clean, an additional acetone clean, followed by an isopropanol wash, was performed. The entire PCB was then coated with approximately 3.3 μm of parylene using a *SCS Labcoater 2* parylene deposition system. A 193 nm excimer laser (*IPG Photonics IX-255*) was then used to open holes in the parylene. The stabilisation energy was selected to be 7 mJ, with a pulse repetition rate of 100 Hz, with a 70 μm by 70 μm spot size, and variable attenuator angle (VAT) of 40 degrees (measured to give a fluence of 2.9 J/cm^2^). The laser was then set to raster over each pad, with an overlap of only 7.1% (65 μm per pulse), for a total of eight passes. The result is a flexible PCB with pads that are now higher than the surrounding traces, and those traces are now insulated by Parylene C.

We note here that some boards were fabricated with the electroplating step bypassed entirely. In those cases, the pillar area was reduced to 425 μm by 425 μm to avoid shorting to board traces, which gives a lower fill factor of only about 18.06% and ultimately a lower ultrasound pressure.

**
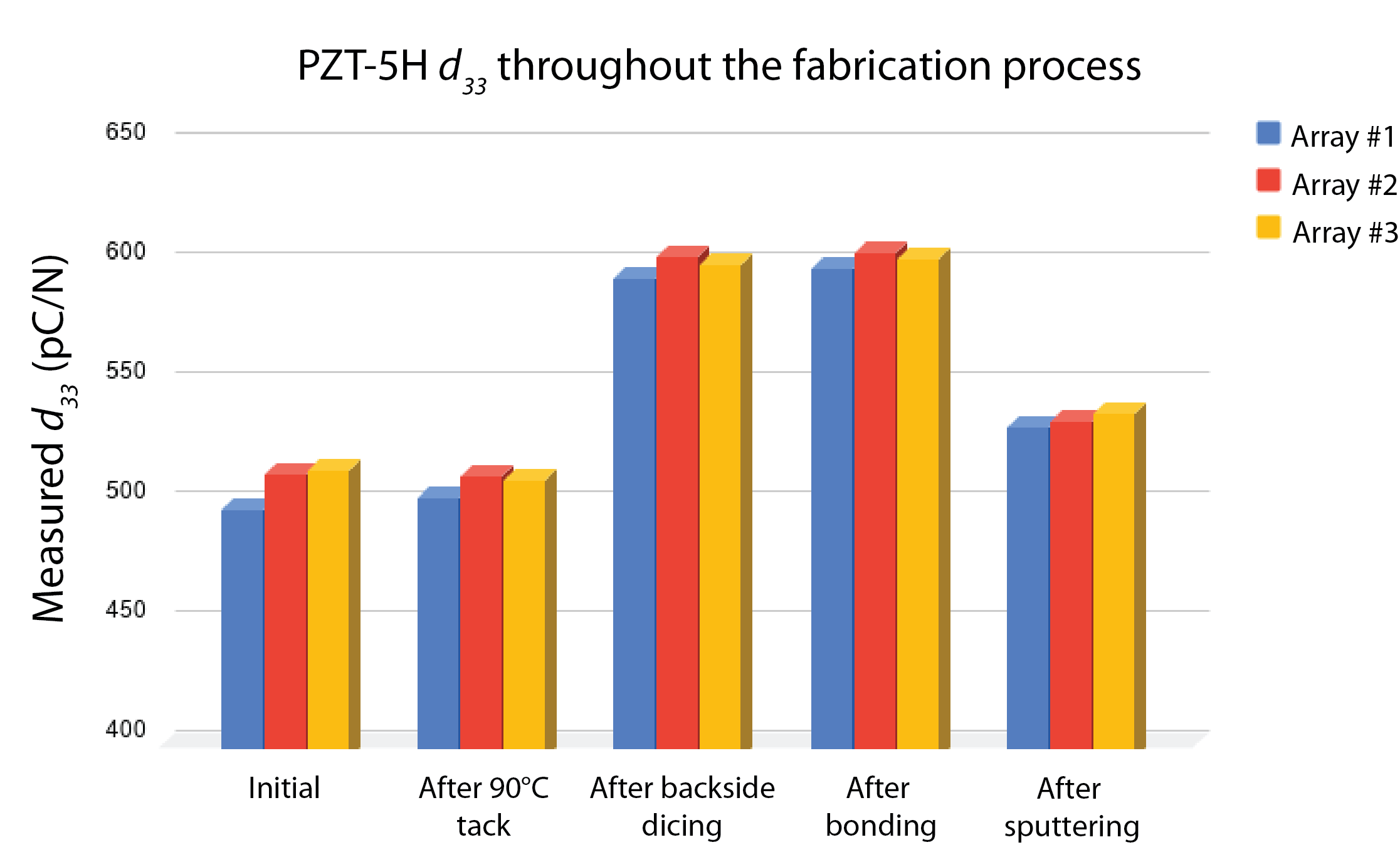
**

**Fig. S3. Measured *d_33_* of three PZT-5H blocks after each major fabrication step.** The piezoelectric coefficient increases throughout the fabrication process for the FlexArray, relative to the as-purchased large PZT crystals.

Piezoelectric crystals are known to return to a non-piezoelectric state after exposure to temperatures above their respective Curie temperatures. This loss in piezoelectricity, due to a permanent realignment of the electric dipoles in the material, is manifested in a drop in the piezoelectric coefficient (*d_33_*). As such, maintaining a low enough temperature throughout the array fabrication process is necessary to maintain electro-mechanical performance. In addition, exposing additional surfaces of the PZT crystal by dicing, and treating the top and bottom surfaces chemically throughout lithography and metal deposition, may also have unwanted effects on the piezoelectric coefficient.

We used a *Piezotest PM300* *d_33_* meter to confirm the piezoelectric coefficient of our PZT materials after each major fabrication step for the FlexArray. The samples were tested with a static force of 10.3 N and a dynamic force of 0.25 N, with a frequency of 110 Hz. As testing individual 825 μm by 825 μm pillars is impractical, a larger 1.6 cm by 1.6 cm piece of the same PZT-5H was used in a shadow process flow mirroring the array processing steps. Three samples underwent processing independently, and each was measured three times after each stage, starting from a baseline measurement before any processing, i.e. fresh after purchasing from the manufacturer. The anisotropic conductive film (ACF) tacking stage was emulated by heating to 90℃ and applying 100 N on the sub-micron bonder for 20 seconds, leading to the same thermal and pressure gradients experienced by the final array. The backside was diced down 500 μm (half of the height) using the same parameters used for making the actual array. Note that the topside was not diced, as the pillars would have come loose without a supporting substrate. The sample was then heated to 160℃ under 375 N of force from the bonder to simulate the bonding procedure, without the substrate. Finally, the sample was sputtered with 10 nm of chromium and 1 μm of copper to test the effects of the top metal deposition on the *d_33_*.

As seen in Fig. S3, the overall piezoelectric properties do not degrade throughout processing. The final array has a *d_33_* that is, on average, 5.25% higher than the initial value before fabrication. Note that the largest increase in the piezoelectric coefficient occurs immediately following the dicing procedure (17.97%). This is unlikely to be caused by thermal effects (which are expected to degrade the performance, as opposed to enhance it), but rather by the structural changes undergone by the material, i.e. the change of the aspect ratio and exposure of additional interfaces. After partial dicing, the resulting PZT may be analogous to a 1-3 piezocomposite, with air serving as the filing medium.


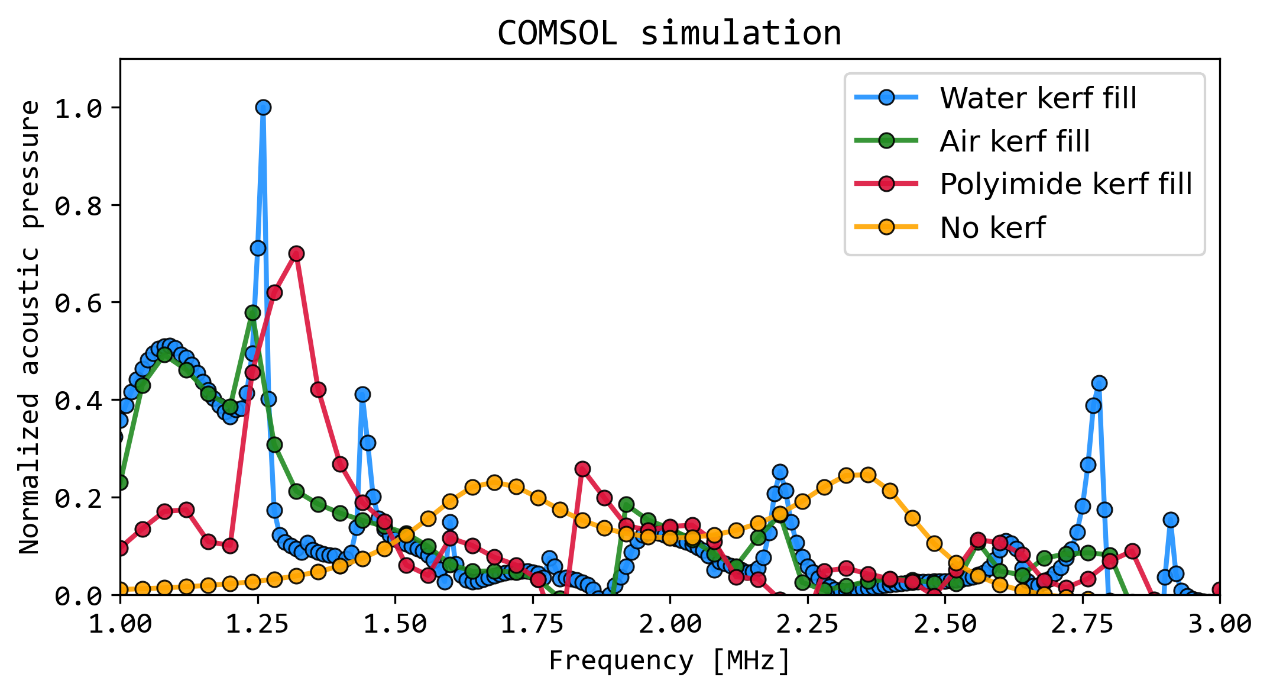


**Fig. S4. COMSOL Multiphysics simulations of the 2D piezoelectric element array.** The modeled PZT pillars have the approximate dimensions of the experimental device pillars, due to fabrication variability from pillar to pillar. A periodic 3D boundary condition was imposed in the Solid Mechanics and Pressure Acoustics modules on the sides of the pillar in the *x* and *y* directions. The flexible substrate properties were chosen to be that of glass-reinforced epoxy (FR-4), commonly used to make PCBs. Different kerf fill materials were simulated, as well as no kerf fill at all (labeled on the plot). Using water or air as the kerf fill material improves the ultrasound coupling from the array to water, at the cost of bandwidth. The associated increase in device flexibility due to the lack of kerf fill, therefore, makes a diced kerf fill-less array design an attractive prospect for highly-flexible 2D ultrasound imagers.

**Supplementary Section 2. Board design and system interconnectivity**

**
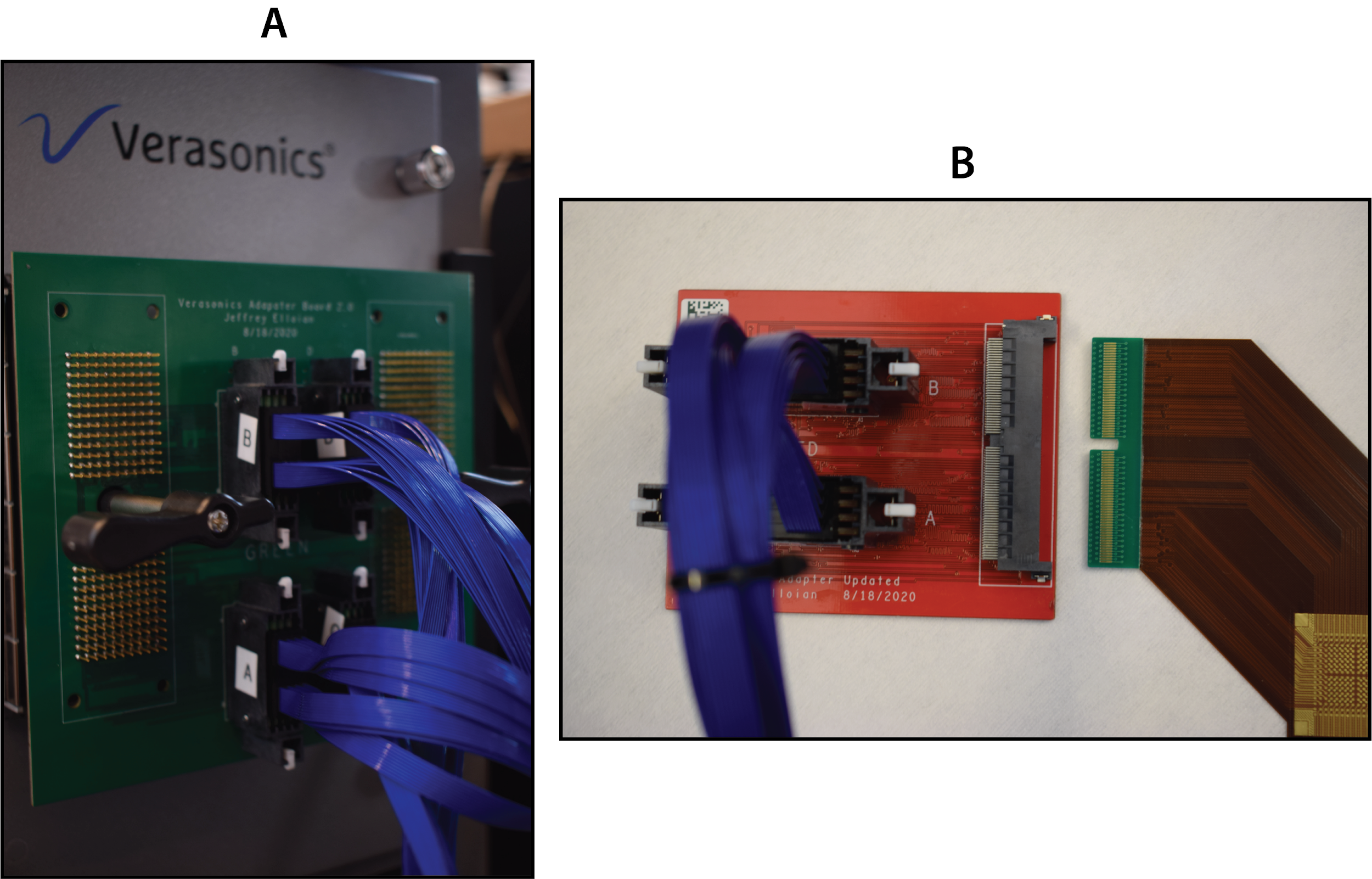
**

**Fig. S5. FlexArray electrical interfaces. a** Custom-made board for interfacing the FlexArray with the *Verasonics Vantage* ultrasound system. **b** The edge-card adapter at the opposite end of the cable allows for direct plug-in to the FlexArray, facilitating rapid exchange of test boards and securing the electrical connections to the device.

**
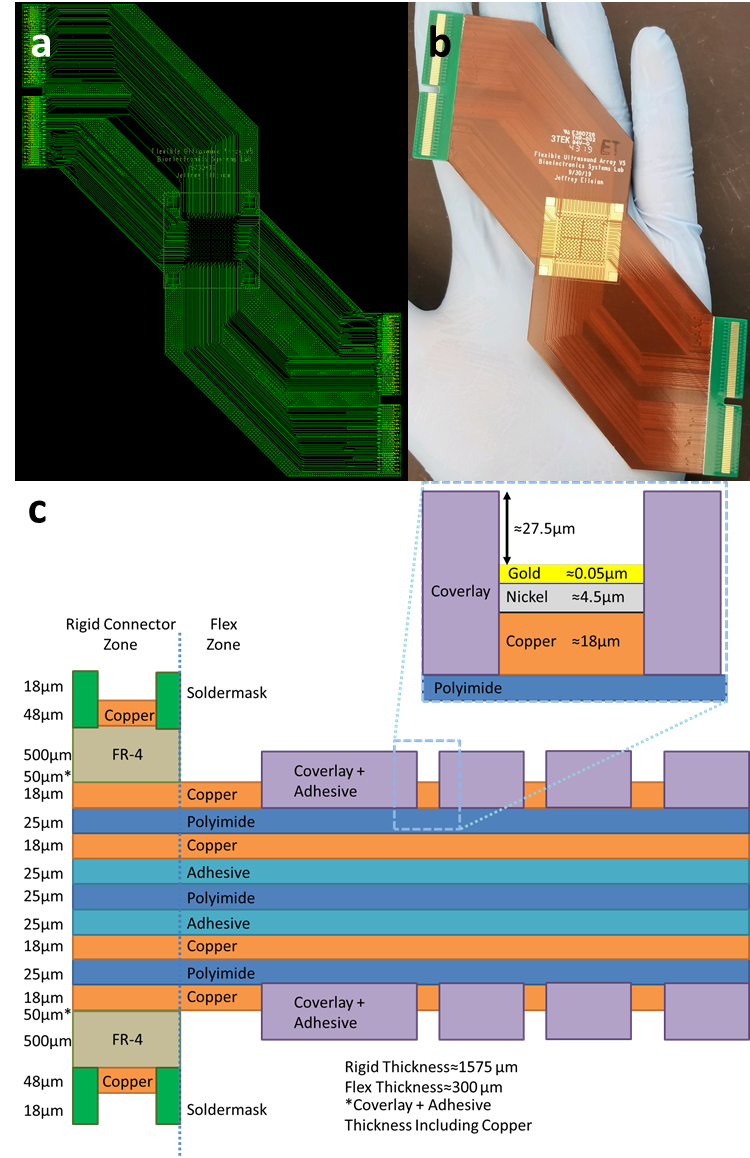
**

**Fig. S6. Flexible PCB Design. a** Computer-aided design of the routing of the top layer of the flexible PCB. **b** FlexArray before processing. **c** Detailed cross-sectional stack-up of the FlexArray prior to processing.

The edge card adapter used here, MEC5-080-01-L-RA-W1-TR, has two rows of 500 μm pitch pads, totaling 160 connections. Of these, 128 per connector are used for carrying signal lines for the elements, leaving 32 for the return ground path. We can calculate the approximate current flowing through the ground network. If we assume we are using PZT-5H and the input impedance is approximately dominated by the capacitance, the input impedance of a single 825 μm × 825μm × 1 mm element at 1.7 MHz is given as:

$$C\approx\frac{\varepsilon_{r}\varepsilon\varepsilon_{0}A}{t}=\frac{3400\varepsilon_{0}(825 \mu m \times825 \mu m)}{1 mm}\approx20.5 pF$$

$$Z_{in}\approx\frac{1}{j\omega C}=4.57 k\Omega$$

To reduce the technological development time, the *Verasonics Vantage 256* system was used as the analog/digital front-end. While providing the design flexibility of being able to control most electronic parameters through MATLAB, it does impose certain restrictions on the design. Most important is the limitation of a maximum of 256 elements. While it is possible to multiplex multiple transducers to each signal pin to increase this number, such an approach is made impractical because it would require active multiplexing circuitry directly on the board, and the *Vantage* system does not support an early trigger signal to allow for the safe separation of transmit and receive signal paths. An overview of the system integration with the *Vantage 256* tool is shown in Fig. S7. The host controller is a PC running MATLAB 2020a, and has the ability to configure a number of internal parameters, allowing for the customisation of the transmitted and received electronic signals. Moreover, the so-called "event" based system in the independent hardware and software sequences allows for asynchronous collection of data and for arbitrary parameters to be set for each rayline. The image reconstruction algorithm is then performed locally, in real-time, on the host controller.

**
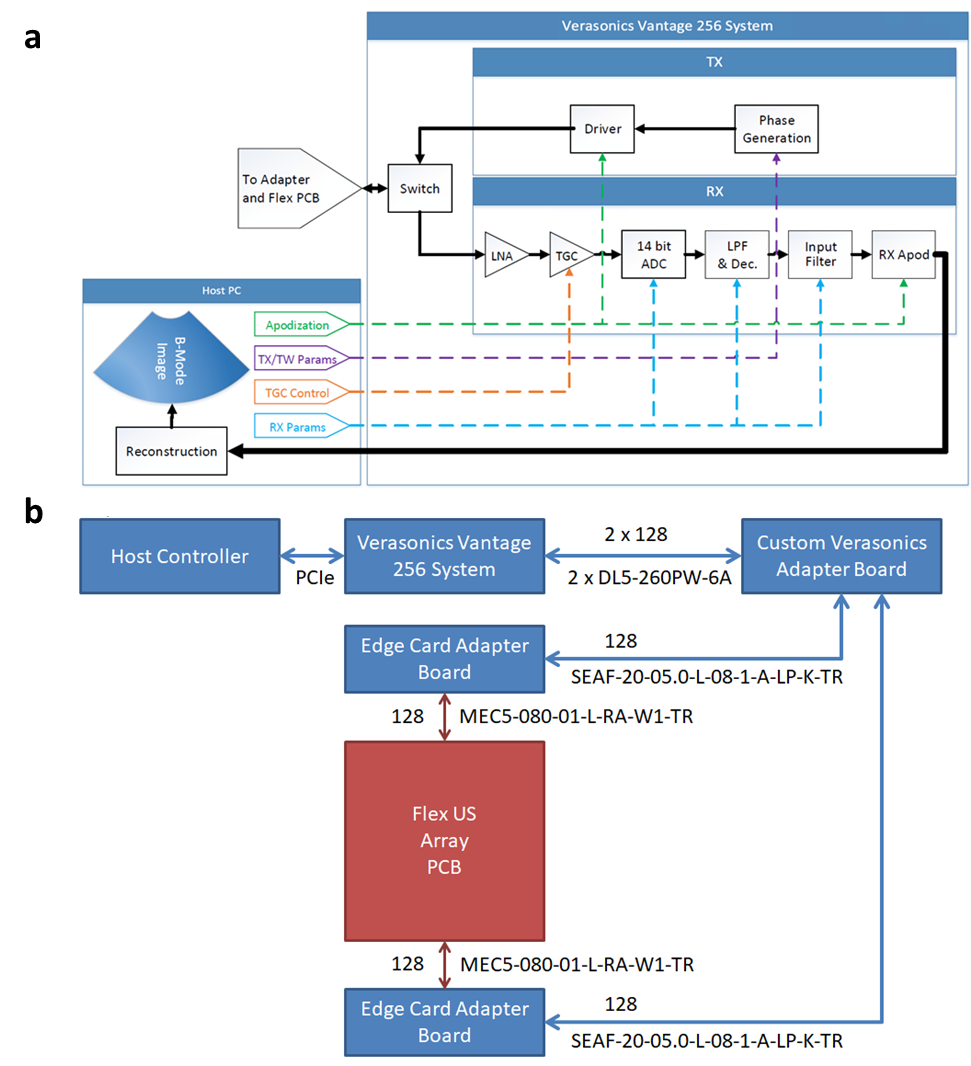
**

**Fig. S7. System interconnectivity**. **a** Block diagram for system and diagram for the *Verasonics Vantage* interface. **b** Connectivity diagram and number of channels for the FlexArray system.

**Supplementary Section 3. Additional simulation results**

In addition to the simulations shown in the main paper, we performed FIELD II simulations to predict the functionality of the transducer array at various radii of curvature; with and without phase correction. As seen in Figs. S8a-d, the phase correction for the B-mode images has an expectedly less-pronounced effect for wider radii of curvature. In Figs. S8e-g, we also present simulation results of beam steering with an unfocused flat array, which we compare to experimental results in Figs. S9e-g.

**
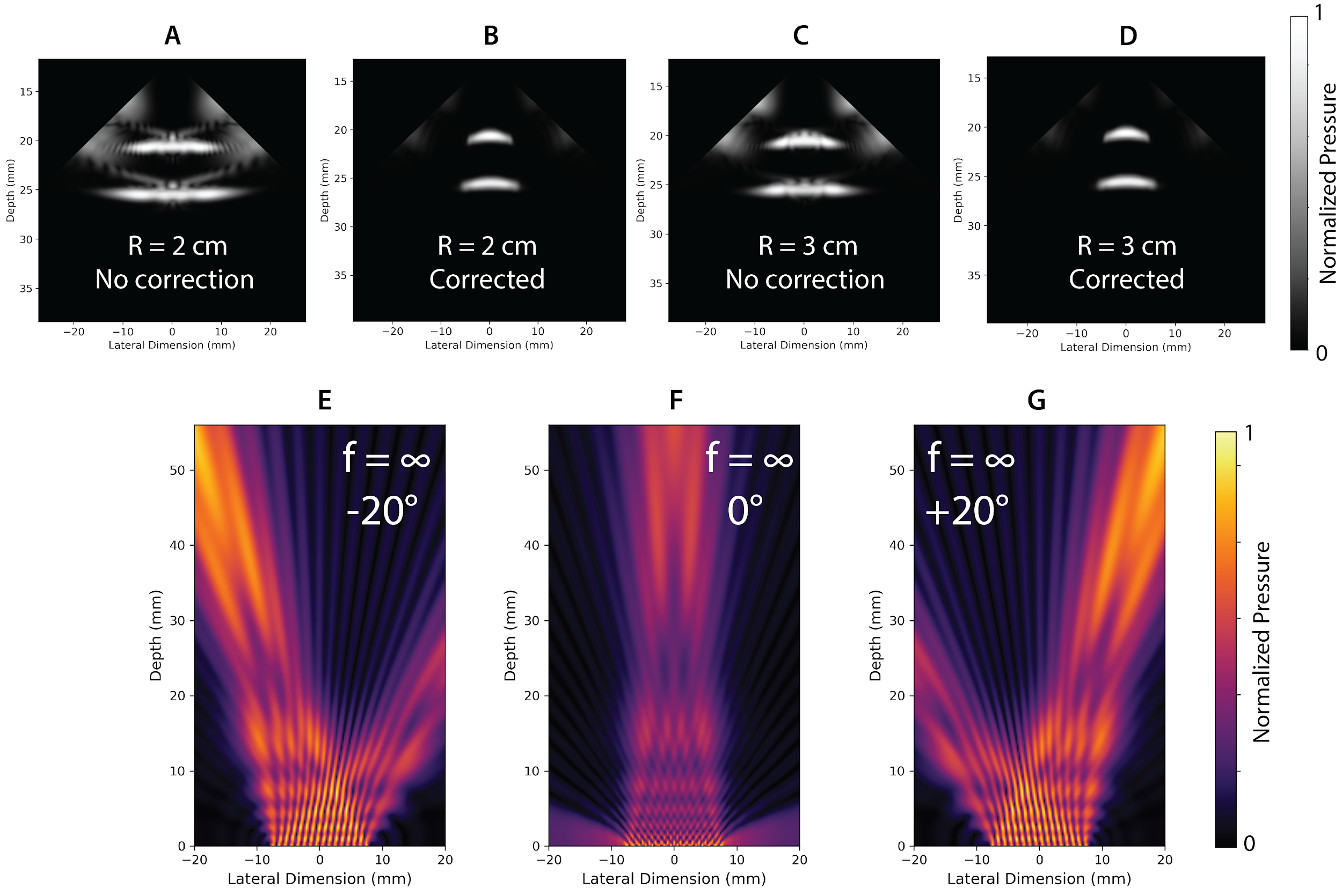
**

**Fig. S8. Simulation of B-mode images at different *R* and of beam steering with focus at infinity. a** Simulated B-mode image without phase correction when *R* = 2 cm. **b** Simulated B-mode image with phase correction when *R* = 2 cm. **c** Simulated B-mode image without phase correction when *R* = 3 cm. **d** Simulated B-mode image with phase correction when *R* = 3 cm. Note that **a**-**d** are all self-normalised to the colorbar on the right. **e** Simulated beam steering pattern with focus at infinity, when the phase shift is - 20°. **f** Simulated beam steering pattern with focus at infinity, when the phase shift is 0°. **g** Simulated beam steering pattern with focus at infinity, when the phase shift is + 20°. Note that **e**-**g** are all self-normalised to the colour bar on the right.

**Supplementary Section 4. Additional experimental results**

The experimental results from the FlexArray in Fig. S9 should be compared directly with the simulated images presented in Fig. S8. We produced additional gelatin phantoms with larger radii of curvature to demonstrate the effect of phase correction as *R* increases, shown in Fig. S8a-d. As expected, as the radius increases the array becomes flatter, and the correction has less of a demonstrable effect on the resulting reconstructed B-mode image. Furthermore, experimental pressure maps for the unfocused (plane wave) beamforming at different steering angles are demonstrated in Figs. S9e-g.

**
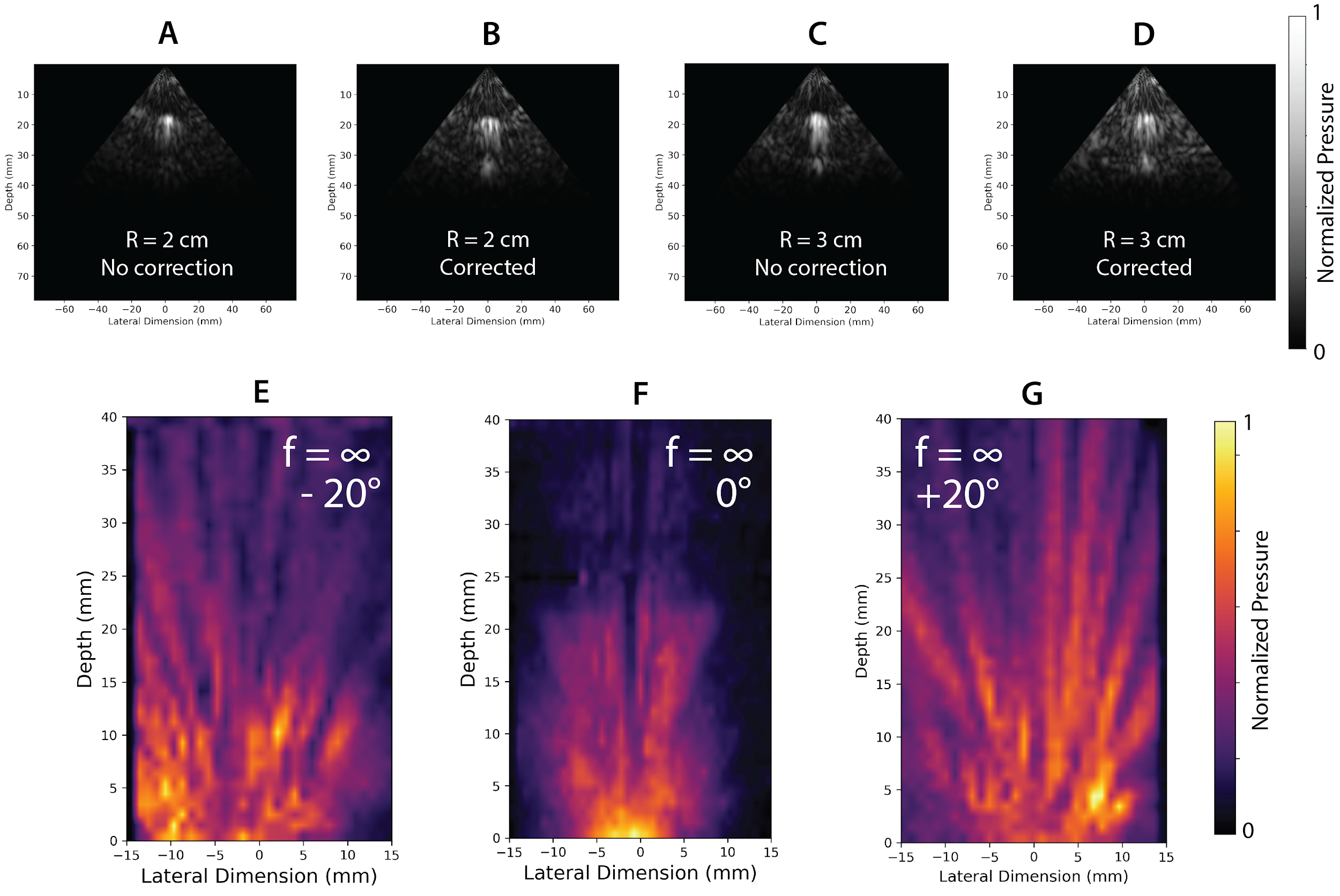
**

**Fig. S9. Experimental results of B-mode images at different *R* and of beam steering with focus at infinity. a** Experimental B-mode image without phase correction taken when *R* = 2 cm. **b** Experimental B-mode image with phase correction when *R* = 2 cm. **c** Experimental B-mode image without phase correction when *R* = 3 cm. **d** Experimental B-mode image with phase correction when *R* = 3 cm. Note that for **a**-**d**, the B-mode images are all compound averaged focal series scans between *f* = 2, 3 and 4 cm and are self-normalised to the colorbar on the right. **e** Experimental beam steering pattern with focus at infinity, when the phase shift is - 20°. **f** Experimental beam steering pattern with focus at infinity, when the phase shift is 0°. **g** Experimental beam steering pattern with focus at infinity, when the phase shift is + 20°. Note that **e**-**g** are all self-normalised to the colour bar on the right.

If the FlexArray is to be conformal to an arbitrary curved surface, it would be of high benefit if it were able to detect the radius of curvature in order to correct for phasing. In the design phase, two paradigms for curvature detection were considered. The first was the use of an external sensor such as a strain gauge mounted along the major axes of the array. Such a method comes with drawbacks of requiring valuable PCB real estate for the sensor, which would also complicate routing. Moreover, additional hardware such as amplifiers would be needed to produce a viable signal, necessitating a regulated power source and additional I/O connections on an otherwise passive array.

The second method was to use the time-of-arrival (TOA) of signals from the transducers. Apodization vectors can be used to only transmit or receive from individual elements or groups of elements on the array. When emitting a pulse from a single PZT pillar, all other transducers can operate in receive mode. Assuming a uniform medium in the near field, *i.e.* a constant phase velocity, *c*, the distance between the transmitter and a given receiver is determined by:

$d = c \times TOA$.

Not all transducers need to be pulsed, provided the radius of curvature is cylindrical in nature. By pulsing at least 2 transducers along an axis, it is possible to create a triangle whose vertices represent the centers of the current location of the pillars. The circle circumscribed by this triangle is the bending radius, *R*, as shown in Fig. S10 below:


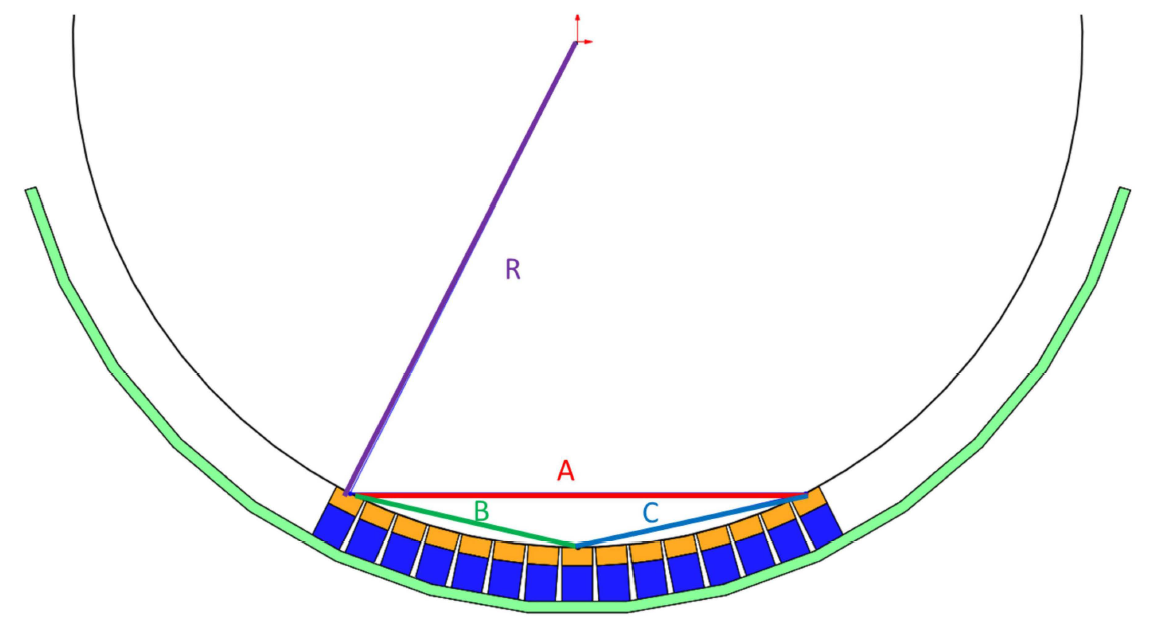


**Fig. S10. Illustration of the time-of-arrival-calculated distances to determine the radius of curvature of the array.**

Given the line segments *A*, *B*, *C* from the calculated distances, it is possible to determine the *R* which would circumscribe the triangle by first finding its area from:

$$k = \sqrt{p(p -A)(p - B)(p - B)}$$

where

$p = \frac{A +B + C}{2}$.

*R* can then be determined by knowing only the distances *A, B, C* using:

$$R = \frac{ABC}{4k} = \frac{ABC}{4\sqrt{\frac{(B-A+C)(A-B+C)(A+B-C)(A+B+C)}{2}}}$$

While only three segments are needed to define the triangle to determine the radius, more points can be used to form unique triangles to confirm the radius. Points that are physically located further apart (producing a higher aspect ratio triangle) will provide a more accurate measurement. With the current device design, it is very difficult to get an accurate estimate for *R*, as this scales sharply with errors in *A, B, C*. We plot the relationship between the error in the determined *R* based on the measured distance error for a family of different true *R* values. As seen in the graph, as the array becomes less curved in places, the error in the determined *R* for a given distance error can be more than 25%. As the array uses 256 transducers, it is difficult to ensure the exact same distance change for all elements around a cylindrical axis, leading to large errors in the estimation of *R* on the real device.


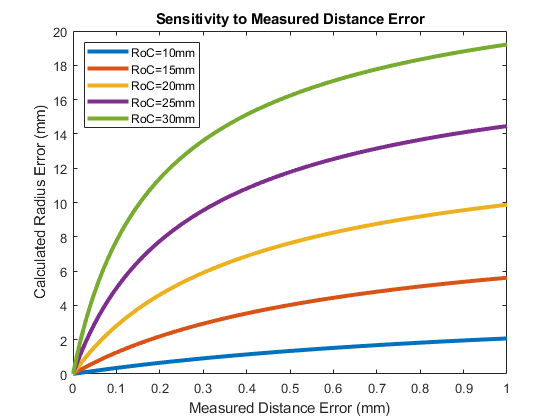


**Fig. S11. Sensitivity-to-measured distance error of the FlexArray radius based on the employed time-of-arrival algorithm.** The less curved the array becomes, the larger the margin of error in detecting its true radius of curvature based on inaccuracies in the determined individual transducer positions.

**
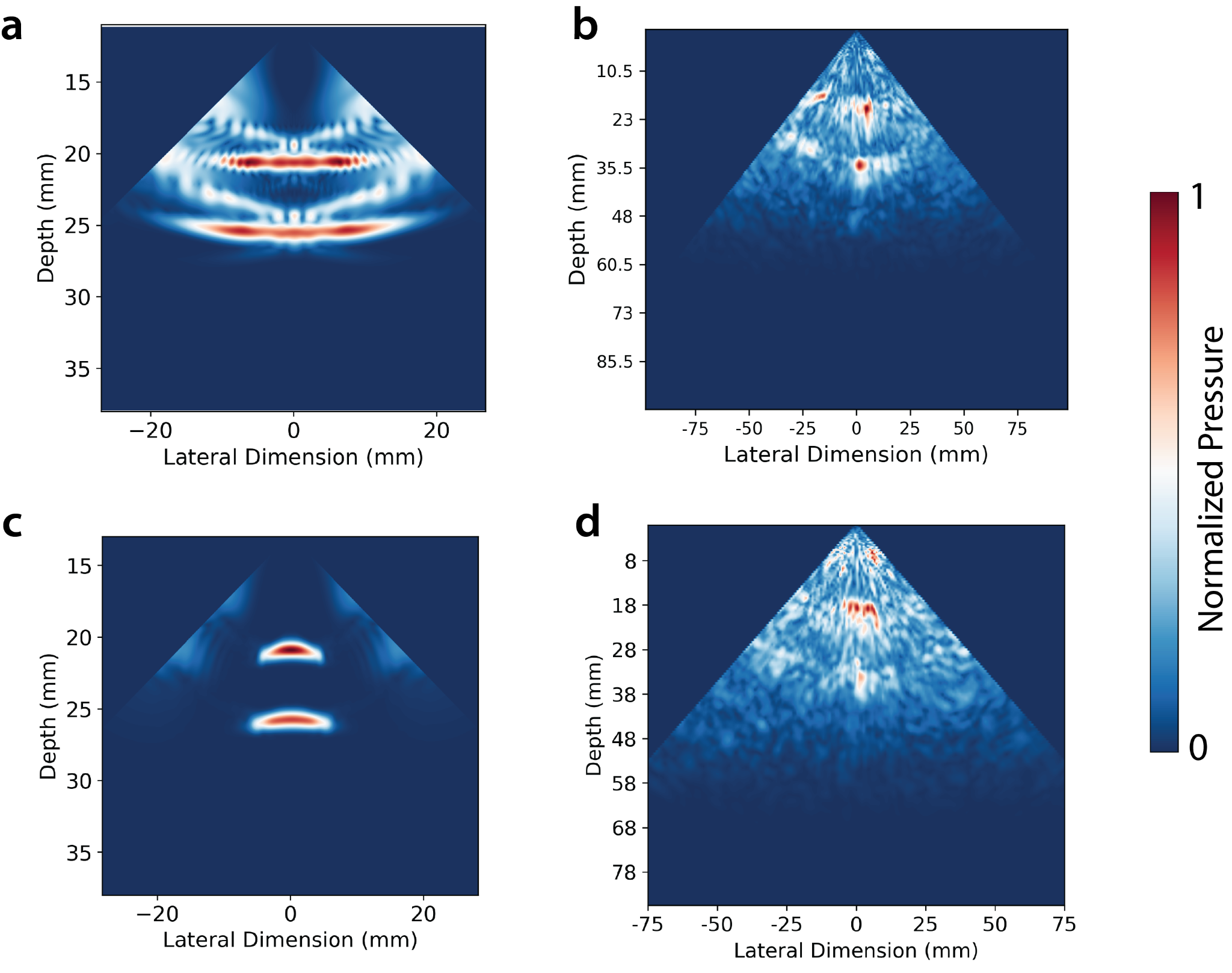
**

**Fig. S12. Simulated and experimental B-mode images from Figure 5 of the main text presented with a divergent colormap for enhanced contrast perception. a** Simulated B-mode image when the radius of curvature is 1.5 cm and phase correction is not implemented. **b** Experimental B-mode image of an air pocket in gelatin captured with the FlexArray when the radius of curvature is 1.5 cm; without phase correction. **c** The same simulation as in **a**, with phase correction implemented. **d** The same B-mode image as in **b**, with implemented phase correction. Note that both images in **b** and **d** are compound averaged focal series images between *f* = 2, 3 and 4 cm.

**Table S1.** **Comparison with similar curved ultrasound arrays in the literature.**

|  | Pashaei et al.^2^ | Cannata et al.^3^ | Hamelmann et al.^4^ | Hu et al.^5^ | **This Work** |
| --- | --- | --- | --- | --- | --- |
| No. of Elements | 64 ⨉ 1 | 64 ⨉ 1 | Staggered 7/8 ⨉ 5 (total: 37) | 10 ⨉ 10 | 16 ⨉ 16 |
| Rigid/Flexible | Flexible | Rigid | Flexible | Flexible | Flexible |
| Technology | Bulk PZT | Bulk 2-2 Composite | Bulk PZT | Bulk 1-3 Composite | Bulk PZT |
| Frequency (MHz) | 5 and 1.3 | 35 | 1.0 | 3.5 | 1.4 |
| Field of View | >0.2 cm ⨉ 2 cm | >0.2 cm ⨉ 1.3 cm | >8 cm ⨉ 15 cm ⨉10 cm | N/A | 12 cm ⨉ 12 cm ⨉ 7 cm |
| Pressure at Focus (kPa V^-1^) | 20 | N/A | N/A | N/A | 15.2 |
| Bandwidth | N/A | 55.0% | N/A | 47.1% | 41.3% |
| Curvature Phase Correction | No | No | No | No | Yes |
| *In vivo* mode | Human | N/A (Wire Phantom) | Chicken | N/A (Wire Phantom) | Human |

**Supplementary Section 5. Derivation of the minimum radius of curvature**

It is difficult to use strain as the metric for determining the minimum radius of curvature, as different parts of the board and the array itself may experience varying strain depending on the topology of the imaged surface and materials used in each section of the finished device. Instead, we directly quantify the minimal radius of curvature before functionality is lost; this failure can be either reversible or irreversible.

Because piezoelectric transducer elements are used, a major limiting factor in the flexibility of the overall array is the physical contact between the tops of the elements during extreme bending. This is a reversible failure mode.

This case may be studied by considering cylindrical bending along a single axis, as demonstrated in Fig. S13, with two elements of width *a*, and fixed pitch *d*. Assuming that *d’* ≅ *d*, that is, there is no buckling or deformation when the array is bent, the arc distance between elements across the bend is preserved. The height, *h*, of the elements can be assumed to be the total height of the pillar. Furthermore, it is assumed that the rectangular elements are rigid, thus width and height do not change with curvature.

In Fig. S13, the primed variables indicate the new flexed distances, which are dependent on the radius of curvature, *R*. For the pillars to physically not be in contact, the arc between elements must be *x'* > 0. To find this arc length, the newly-formed segment, *R’*, can be used. *R’* is given by the hypotenuse of the right triangle formed by the pillar and the radius of curvature:

$$R'=\sqrt{\frac{a^{2}}{4}+(R-h)^{2}}$$

The inner arc length, *x’*, can be defined as:

$$x'=\theta_{2}R'$$

while the outer arc length, *d’*, which is described by θ_2_ can be found by subtracting away the arc segments labeled *y’* in Fig. S10:

$$y'=\theta_{1}R$$

While the angles θ_1_ and θ_2_ are not explicitly known, they relate the arc segment given by *d'-2y'* to the radius of curvature:

$$\theta_{2}=\frac{d'-2y'}{R}$$

and

$$\theta_{1}=\frac{0.5a}{R-h}$$

where *a* is the width of the transducer elements. An expression for the arc lengths can then be found:

$$x'=\frac{R'(d'-2y')}{R}$$

and

$$y'=\frac{R(0.5a)}{R-h}$$

Substituting the expressions for $y'$ and $R^{'}$ into the definition of $x^{'}$ provides the resulting arc length and flexibility condition in terms of the known design variables (*a*, *d* , *h* and *R*):

$$x'=\frac{1}{R}\sqrt{\frac{a^{2}}{4}+(R-h)^{2}}\left( d-2R{tan}^{-1}\left( \frac{0.5a}{R-h} \right) \right)>0$$

This transcendental inequality cannot be solved analytically. However, most of these values are fixed because of physical constraints in real devices. For example, the element pitch is limited by the compromise between grating lobes and resolution while the element height is restricted by the resonant frequency of operation. Element width, in turn, has an effect on output pressure, and thus the SNR; as such, it needs to be optimised to a single value. $x'$ can, therefore be solved numerically for realistic device cases. In Fig. S13, we compute the theoretical minimum radius of curvature that is achievable with an element pitch of 1 mm. Note that these are geometrical limits, and may be further limited by the rigidity of the chosen substrate.

For all cases tested in this work, *a <* 825 μm and *h* > 1 mm, such that the theoretical minimum radius of curvature achievable with the FlexArray is *R <* 1 cm.


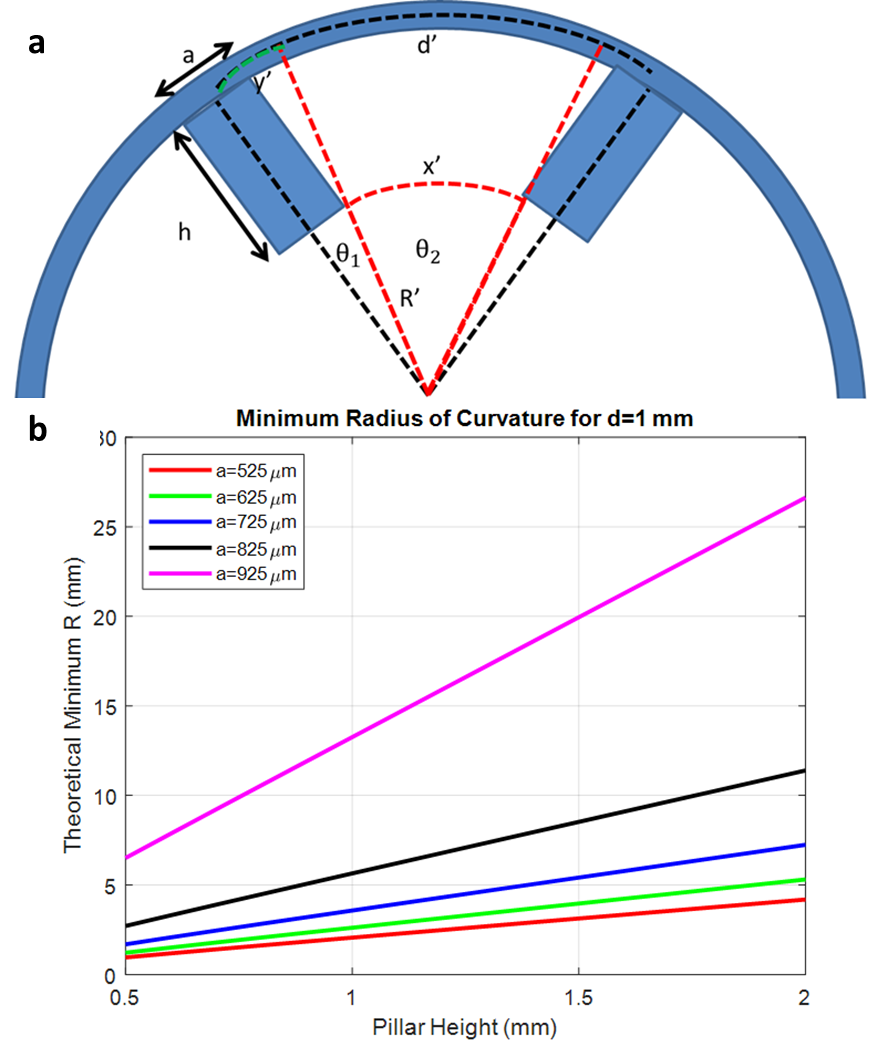


**Fig. S13. Geometric derivation of the minimum radius of curvature. a** Diagram presenting the key variables which change when flexing in a single axis. **b** Graph of the theoretical minimum radius of curvature for various fixed element pitch values.


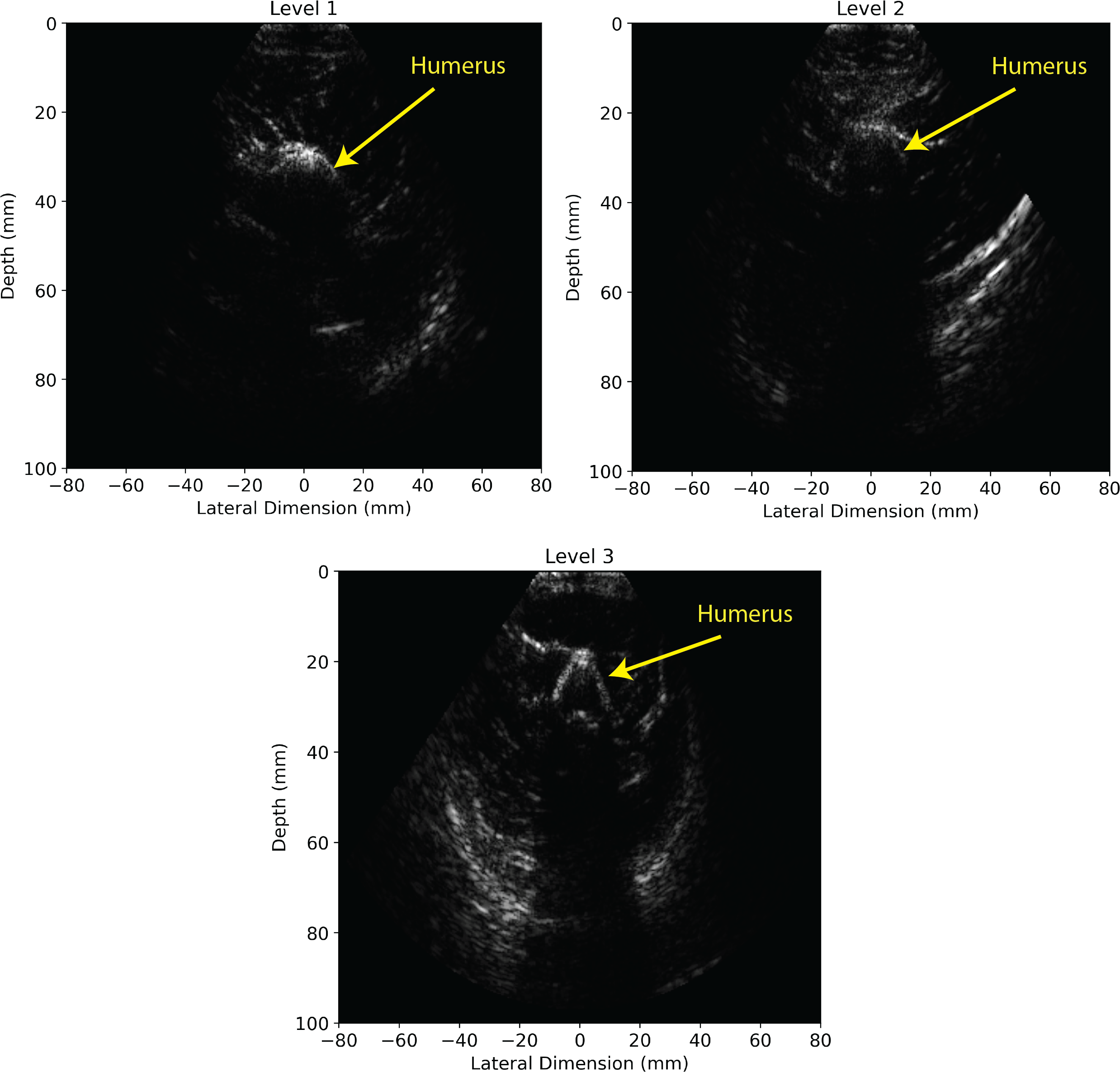


**Fig. S14. Sonograms of a human humerus taken at the three different scan locations with a commercial probe.** The top surface of the bone is visible at the 2-2.5 cm depth region in all three scans. The frequency of the probe used here was 2.5 MHz.


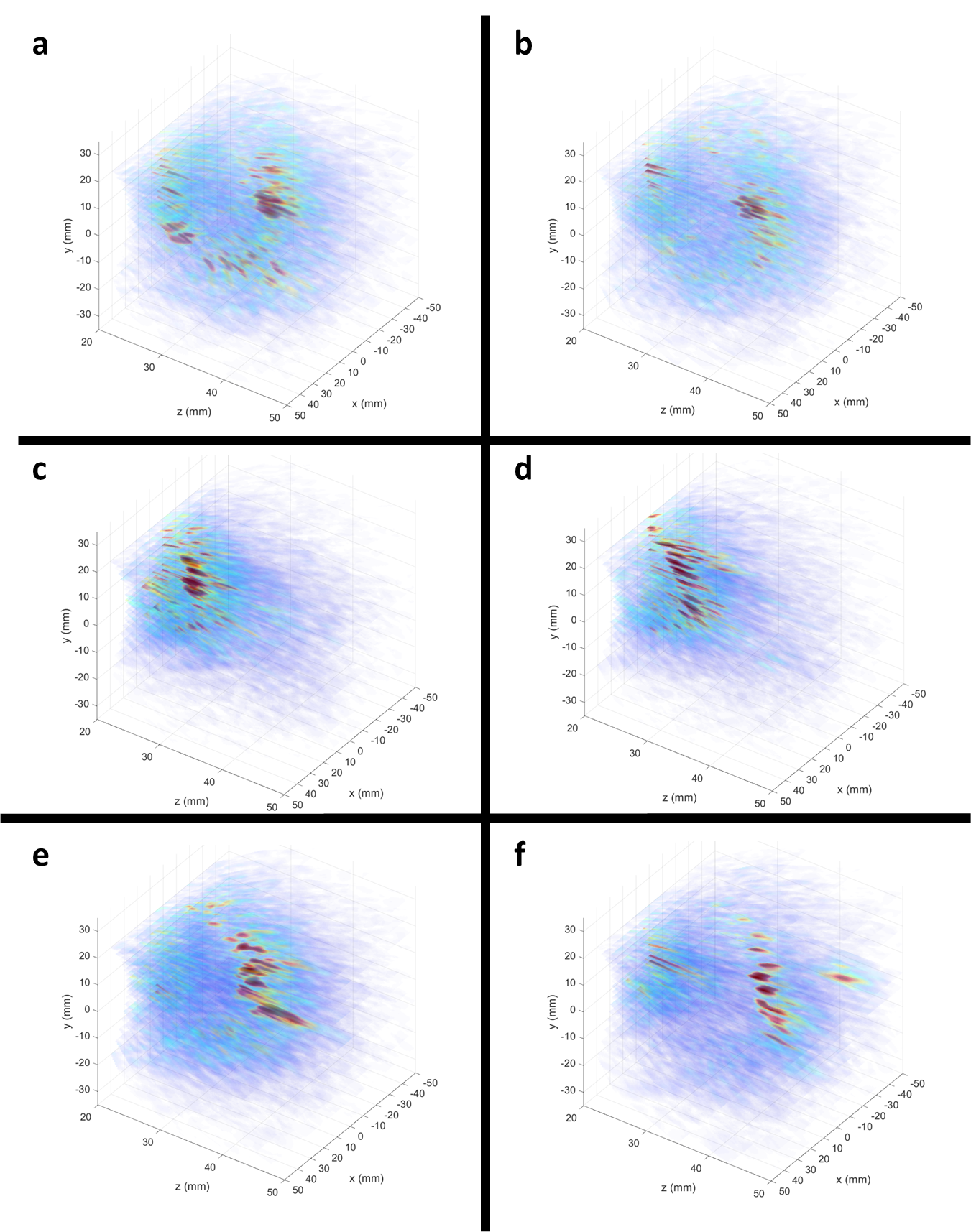


**Fig. S15. 3D B-mode images of a human humerus.** Combined slices of 16 *y-z* scan angles to produce 3D images of the humerus at (**a**,**b**) Level 1, (**c**,**d**) Level 2 and (**e**,**f**) Level 3 measurement locations. (**a,c,e**) The resulting images using traditional phasing and reconstruction, regardless of the radius. (**b,d,f**) The same position on the arm, now imaged using the corrected radius of curvature. The humeral shaft is seen to be confined to a more localised volume in the phase-corrected reconstructed sonograms.

**Supplementary References**

1. Z. Gubinyi, C. Batur, A. Sayir, F. Dynys, Electrical properties of PZT piezoelectric ceramic at high temperatures. *J. Electroceram.* **20**(2), 95-105 (2008)
2. V. Pashaei, P. Dehghanzadeh, G. Enwia, M. Bayat, S. J. Majerus, S. Mandal, Flexible body-conformal ultrasound patches for image-guided neuromodulation. *IEEE T. Biomed. Circ. S.* **14**, 1–1 (2019)
3. J. M. Cannata, J. A. Williams, Q. Zhou, T. A. Ritter, K. K. Shung, Development of a 35-MHz piezo-composite ultrasound array for medical imaging. *IEEE Trans. Ultrason. Ferroelectr. Freq. Control* **53**, 224-236 (2006)
4. P. Hamelmann, M. Mischi, A. F. Kolen, J. O. Van Laar, R. Vullings, J. W. Bergmans, Fetal heart rate monitoring implemented by dynamic adaptation of transmission power of a flexible ultrasound transducer array. *Sensors (Basel)* **19**, 1195 (2019)
5. H. Hu, X. Zhu, C. Wang, L. Zhang, X. Li, S. Lee, Z. Huang, R. Chen, Z. Chen, C. Wang, Y. Gu, Y. Chen, Y. Lei, T. Zhang, N. Kim, Y. Guo, Y. Teng, W. Zhou, Y. Li, A. Nomoto, S. Sternini, Q. Zhou, M. Pharr, F. Lanza di Scalea, S. Xu, Stretchable ultrasonic transducer arrays for three-dimensional imaging on complex surfaces. *Sci. Adv.* **4**:eaar3979 (2018)
