# Supplementary material for: Flexible ultrasound transceiver array for non-invasive surface-conformable imaging enabled by geometric phase correction
Source: Sci Rep. 2022 Sep 28;12:16184. doi: 10.1038/s41598-022-20721-7 (PMC9519534; doi:10.1038/s41598-022-20721-7)
Supplement: Supplementary file 3 — Supplementary Information 2. [file 41598_2022_20721_MOESM3_ESM.docx]

Supplementary Materials

Supplementary Information included as a separate file.

Contents: Cross-sectional fabrication flow for the FlexArray, detailed descriptions of the fabrication of the piezoelectric pillar array, measured changes to the d33 of three PZT-5H blocks after each major fabrication step, COMSOL simulations, description of the electrical interfaces to the FlexArray, discussion of the flexible PCB design, discussion of system interconnectivity, simulation of B-mode images at different R and of beam steering with focus at infinity, experimental results of B-mode images at different R and of beam steering with focus at infinity, geometric derivation of the minimum radius of curvature, 3D compound images of the human humerus.

Supplementary Movie of the in-vivo humerus B-mode scan in real time during triceps contraction.
